# Supplementary material for: Estimating causes of community death of adults in Myanmar from a nationwide population sample: Application of verbal autopsy
Source: PLOS Glob Public Health. 2023 Nov 1;3(11):e0002426. doi: 10.1371/journal.pgph.0002426 (PMC10619871; doi:10.1371/journal.pgph.0002426)
Supplement: S1 Text — (DOCX) [file pgph.0002426.s001.docx]

**S1 Text: Estimation of the under-five mortality rate as input into the empirical completeness method**

An input into the empirical completeness method is an estimate of the under-five mortality rate of the state within which the township is located, calculated as an average of the figure in the 2014 Population Census and the 2015-2016 Demographic and Health Survey, and then scaled to the national estimate from the Inter-agency Group for Child Mortality Estimation (IGME) (21-23) From these calculations, the under-five mortality rate is almost identical between the 42 townships (45·4) and the national level (44·6). VAs as a percentage of community deaths was calculated by subtracting hospital deaths from estimated total deaths.
