# Supplementary material for: Estimating causes of community death of adults in Myanmar from a nationwide population sample: Application of verbal autopsy
Source: PLOS Glob Public Health. 2023 Nov 1;3(11):e0002426. doi: 10.1371/journal.pgph.0002426 (PMC10619871; doi:10.1371/journal.pgph.0002426)
Supplement: S1 Table — (DOCX) [file pgph.0002426.s002.docx]

**S1 Table: Projected mid-year population in Myanmar in the 42 townships 2018 and 2019**

| **Location** | | **2018** | | | **2019** | | |
| --- | --- | --- | --- | --- | --- | --- | --- |
| **State/Region** | **Township** | **Both Sexes** | **Male** | **Female** | **Both Sexes** | **Male** | **Female** |
| Total | | 8,289,112 | 3,943,375 | 4,345,737 | 8,364,513 | 3,975,026 | 4,389,487 |
| Kachin | Myitkyina | 345,637 | 166,679 | 178,958 | 352,109 | 169,813 | 182,296 |
|  | Bamaw | 148,209 | 72,579 | 75,630 | 151,039 | 73,966 | 77,073 |
| Kayah | Loikaw | 141,418 | 691,40 | 72,277 | 144,501 | 70,587 | 73,914 |
|  | Demoeso | 86,960 | 42,541 | 44,419 | 88,762 | 43,388 | 45,374 |
| Kayin | Hpan-an | 430,892 | 206,420 | 224,472 | 433,531 | 207,272 | 226,259 |
|  | Myawaddy | 212,254 | 107,787 | 104,467 | 213,078 | 108,070 | 105,009 |
| Chin | Falam | 51,410 | 25,147 | 26,262 | 51,991 | 25,403 | 26,588 |
|  | Mindut | 45,620 | 21,083 | 24,537 | 46,101 | 21,277 | 24,824 |
| Sagaing* | Ayartaw | 162,028 | 73,326 | 88,702 | 163,370 | 73,868 | 89,503 |
|  | Shwebo | 277,352 | 128,149 | 149,203 | 279,585 | 129,088 | 150,498 |
|  | Myinmu | 111,653 | 50,897 | 60,757 | 112,669 | 51,306 | 61,363 |
|  | Myaung | 111,109 | 49,577 | 61,532 | 112,136 | 49,988 | 62,149 |
|  | Yinmarpin | 146,838 | 68,322 | 78,516 | 147,951 | 68,804 | 79,147 |
|  | Sarlingyi | 126,731 | 57,794 | 68,937 | 127,794 | 58,226 | 69,568 |
|  | Palae | 149,522 | 69,225 | 80,297 | 150,670 | 69,712 | 80,957 |
| Tanintharyi | Dawei | 154,063 | 73,780 | 80,283 | 155,669 | 74,470 | 81,199 |
|  | Myeik | 296,802 | 145,214 | 151,588 | 299,449 | 146,408 | 153,041 |
| Bago | Daikoo | 204,285 | 96,713 | 107,572 | 204,510 | 96,711 | 107,799 |
|  | Nattalin | 175,393 | 83,113 | 92,280 | 176,065 | 83,324 | 92,741 |
| Magway* | Myothit | 160,077 | 73,847 | 86,230 | 159,828 | 73,605 | 86,223 |
|  | Salin | 237,298 | 108,532 | 128,767 | 237,143 | 108,263 | 128,880 |
|  | Kanma | 75,885 | 35,863 | 40,022 | 75,914 | 35,799 | 40,115 |
|  | Yesagyo | 217,102 | 94,416 | 122,687 | 217,076 | 94,208 | 122,869 |
|  | Seikphyu | 103,101 | 46,768 | 56,333 | 102,948 | 46,622 | 56,326 |
| Mandalay | Nyaung-Oo | 251,314 | 113,890 | 137,424 | 253,901 | 114,887 | 139,014 |
|  | Wundwin | 240,493 | 110,576 | 129,917 | 242,961 | 111,571 | 131,390 |
| Mon* | Thanphyuzayet | 166,244 | 78,227 | 88,017 | 165,384 | 77,575 | 87,809 |
|  | Paung | 213,006 | 101,398 | 111,608 | 211,770 | 100,510 | 111,261 |
| Rakhine | Ponagyun | 194,693 | 91,649 | 103,044 | 195,587 | 91,978 | 103,609 |
|  | Kyaukphyu | 172,186 | 83,195 | 88,991 | 173,118 | 83,511 | 89,606 |
| Yangon | Thanlin | 293,857 | 142,140 | 151,718 | 300,432 | 145,098 | 155,334 |
|  | Thonegwa | 173,164 | 82,195 | 90,969 | 177,134 | 83,938 | 93,196 |
| Shan (South) | Kalaw | 200,034 | 99,841 | 100,192 | 203,080 | 101,309 | 101,771 |
|  | Loilem | 133,948 | 64,902 | 69,046 | 135,967 | 65,824 | 70,143 |
| Shan (East) | Kyinetone | 184,904 | 94,255 | 90,649 | 187,856 | 95,682 | 92,174 |
|  | Tachileik | 191,070 | 96,863 | 94,207 | 194,105 | 98,339 | 95,766 |
| Shan (north) | Lashio | 348,041 | 170,055 | 177,986 | 353,497 | 172,577 | 180,921 |
|  | Kyaukme | 185,754 | 88,990 | 96,764 | 188,764 | 90,349 | 98,415 |
| Ayeyarwady | Pathein | 385,999 | 186,653 | 199,345 | 386,089 | 186,549 | 199,540 |
|  | Hinthada | 344,462 | 161,899 | 182,563 | 345,028 | 161,997 | 183,031 |
| NPT | Tatkon | 235,647 | 112,177 | 123,470 | 239,861 | 114,072 | 125,789 |
|  | Pyinmana | 202,657 | 97,558 | 105,099 | 206,088 | 99,082 | 107,006 |
