# Supplementary material for: Estimating causes of community death of adults in Myanmar from a nationwide population sample: Application of verbal autopsy
Source: PLOS Glob Public Health. 2023 Nov 1;3(11):e0002426. doi: 10.1371/journal.pgph.0002426 (PMC10619871; doi:10.1371/journal.pgph.0002426)
Supplement: S2 Table — (DOCX) [file pgph.0002426.s003.docx]

**S2 Table: Population age distribution (age 12+) of 42 townships for each sex**

| Age | Male | Female |
| --- | --- | --- |
| 12 - 14 | 8.6% | 7.4% |
| 15 - 19 | 12.0% | 11.2% |
| 20 - 24 | 10.8% | 10.5% |
| 25 - 29 | 10.5% | 10.2% |
| 30 - 34 | 10.1% | 9.8% |
| 35 - 39 | 9.3% | 9.1% |
| 40 - 44 | 8.5% | 8.5% |
| 45 - 49 | 7.5% | 7.7% |
| 50 - 54 | 6.5% | 6.8% |
| 55 - 59 | 5.2% | 5.6% |
| 60 - 64 | 4.0% | 4.4% |
| 65 - 69 | 2.7% | 3.0% |
| 70 - 74 | 1.7% | 2.1% |
| 75 - 79 | 1.3% | 1.7% |
| 80 + | 1.2% | 1.8% |
| Total | 100% | 100% |
