# Supplementary material for: Estimating causes of community death of adults in Myanmar from a nationwide population sample: Application of verbal autopsy
Source: PLOS Glob Public Health. 2023 Nov 1;3(11):e0002426. doi: 10.1371/journal.pgph.0002426 (PMC10619871; doi:10.1371/journal.pgph.0002426)
Supplement: S3 Table — (DOCX) [file pgph.0002426.s004.docx]

**S3 Table: Completeness of verbal autopsy as a proportion of community deaths (by township)**

| **Location** | | **2018** | | | **2019** | | |
| --- | --- | --- | --- | --- | --- | --- | --- |
| **State/Region** | **Township** | **Both Sexes** | **Male** | **Female** | **Both Sexes** | **Male** | **Female** |
| Total | | 67% | 76% | 60% | 71% | 81% | 62% |
| Kachin | Myitkyina | 62% | 84% | 46% | 65% | 87% | 49% |
|  | Bamaw | 81% | 98% | 67% | 88% | 98% | 79% |
| Kayah | Loikaw | 62% | 76% | 55% | 79% | 112% | 63% |
|  | Demoeso | 78% | 86% | 70% | 67% | 71% | 66% |
| Kayin | Hpan-an | 82% | 84% | 79% | 90% | 99% | 79% |
|  | Myawaddy | 66% | 72% | 64% | 82% | 92% | 73% |
| Chin | Falam | 61% | 69% | 54% | 45% | 57% | 36% |
|  | Mindut | 51% | 66% | 40% | 45% | 50% | 44% |
| Sagaing* | Ayartaw | 66% | 75% | 59% | 67% | 74% | 62% |
|  | Shwebo | 62% | 74% | 52% | 69% | 79% | 58% |
|  | Myinmu | 80% | 88% | 68% | 87% | 92% | 77% |
|  | Myaung | 86% | 90% | 84% | 81% | 86% | 76% |
|  | Yinmarpin | 72% | 83% | 60% | 65% | 73% | 56% |
|  | Sarlingyi | 77% | 88% | 63% | 81% | 87% | 73% |
|  | Palae | 60% | 67% | 56% | 67% | 74% | 60% |
| Tanintharyi | Dawei | 51% | 57% | 48% | 76% | 101% | 60% |
|  | Myeik | 68% | 74% | 61% | 87% | 101% | 77% |
| Bago | Daikoo | 77% | 85% | 70% | 76% | 81% | 71% |
|  | Nattalin | 81% | 87% | 72% | 74% | 77% | 70% |
| Magway* | Myothit | 61% | 70% | 54% | 67% | 74% | 62% |
|  | Salin | 62% | 69% | 57% | 57% | 62% | 55% |
|  | Kanma | 66% | 75% | 57% | 66% | 71% | 62% |
|  | Yesagyo | 68% | 79% | 59% | 66% | 76% | 58% |
|  | Seikphyu | 73% | 81% | 65% | 77% | 85% | 68% |
| Mandalay | Nyaung-Oo | 135% | 148% | 115% | 96% | 102% | 85% |
|  | Wundwin | 77% | 84% | 68% | 79% | 87% | 67% |
| Mon* | Thanphyuzayet | 94% | 94% | 93% | 93% | 96% | 91% |
|  | Paung | 101% | 104% | 96% | 93% | 97% | 89% |
| Rakhine | Ponagyun | 28% | 30% | 31% | 17% | 20% | 18% |
|  | Kyaukphyu | 52% | 67% | 41% | 63% | 80% | 51% |
| Yangon | Thanlin | 83% | 101% | 72% | 84% | 104% | 70% |
|  | Thonegwa | 71% | 73% | 72% | 59% | 61% | 60% |
| Shan (South) | Kalaw | 40% | 44% | 38% | 52% | 63% | 41% |
|  | Loilem | 41% | 50% | 36% | 48% | 59% | 40% |
| Shan (East) | Kyinetone | 36% | 42% | 31% | 31% | 34% | 31% |
|  | Tachileik | 38% | 45% | 33% | 35% | 38% | 35% |
| Shan (north) | Lashio | 31% | 41% | 27% | 53% | 67% | 44% |
|  | Kyaukme | 65% | 72% | 59% | 63% | 71% | 55% |
| Ayeyarwady | Pathein | 37% | 43% | 36% | 42% | 56% | 38% |
|  | Hinthada | 63% | 70% | 58% | 74% | 90% | 60% |
| NPT | Tatkon | 52% | 60% | 48% | 54% | 59% | 51% |
|  | Pyinmana | 61% | 84% | 43% | 63% | 70% | 58% |

* Pilot sites
