# Supplementary material for: Estimating causes of community death of adults in Myanmar from a nationwide population sample: Application of verbal autopsy
Source: PLOS Glob Public Health. 2023 Nov 1;3(11):e0002426. doi: 10.1371/journal.pgph.0002426 (PMC10619871; doi:10.1371/journal.pgph.0002426)
Supplement: S4 Table — (DOCX) [file pgph.0002426.s005.docx]

**S4 Table: CSMFs (%) leading causes of death in adult VA compared to GBD 2019**

**Table A CSMFs (%), leading 15 causes of death in adult males (VA 2018/2019 and GBD 2019)**

| VA | % | GBD | % |
| --- | --- | --- | --- |
| Stroke | 24 | Stroke | 20 |
| Cirrhosis | 14 | Chronic Respiratory | 12 |
| Chronic Respiratory | 10 | Ischemic Heart Disease | 10 |
| Ischemic Heart Disease | 10 | Cirrhosis | 8 |
| Diabetes | 5 | Diabetes | 6 |
| Other Non-communicable Diseases | 5 | Other Non-communicable Diseases | 5 |
| Pneumonia | 4 | Pneumonia | 4 |
| Tuberculosis | 3 | Tuberculosis | 4 |
| Chronic Kidney Disease | 3 | Other Cardiovascular Diseases | 4 |
| AIDS | 2 | Lung Cancer | 4 |
| Prostate Cancer | 2 | Other Cancers | 3 |
| Leukemia/Lymphomas | 2 | Chronic kidney disease | 3 |
| Lung Cancer | 2 | Road Traffic | 2 |
| Esophageal Cancer | 2 | Falls | 2 |
| Road Traffic | 2 | Leukemia/Lymphoma | 2 |

**Table B CSMFs (%), leading 15 causes of death in adult females (VA 2018/2019 and GBD 2019)**

| **VA** | **%** | **GBD** | **%** |
| --- | --- | --- | --- |
| Stroke | 25 | Stroke | 25 |
| Ischemic Heart Disease | 16 | Ischemic Heart Disease | 11 |
| Chronic Respiratory | 12 | Chronic Respiratory | 10 |
| Diabetes | 8 | Diabetes | 6 |
| Other Non-communicable Diseases | 4 | Other Non-communicable Diseases | 6 |
| Pneumonia | 4 | Other Cardiovascular Diseases | 4 |
| Chronic Kidney Disease | 3 | Other Cancers | 4 |
| Cirrhosis | 3 | Pneumonia | 4 |
| Cervical Cancer | 2 | Chronic kidney disease | 3 |
| Breast Cancer | 2 | Cirrhosis | 3 |
| Leukemia/Lymphomas | 2 | TB | 3 |
| Tuberculosis | 2 | Breast Cancer | 3 |
| Diarrhoea/Dysentery | 2 | Leukemia/Lymphoma | 2 |
| Esophageal Cancer | 1 | Lung Cancer | 2 |
| Other cancers | 1 | AIDS | 2 |
