# Supplementary material for: Estimating causes of community death of adults in Myanmar from a nationwide population sample: Application of verbal autopsy
Source: PLOS Glob Public Health. 2023 Nov 1;3(11):e0002426. doi: 10.1371/journal.pgph.0002426 (PMC10619871; doi:10.1371/journal.pgph.0002426)
Supplement: S5 Table — (DOCX) [file pgph.0002426.s006.docx]

**S5 Table: Undetermined cause of death (adult) by State/Region (2018/2019 VA data combined)**

|  | **Ayeyarwady** | **Bago** | **Chin** | **Kachin** | **Kayah** | **Kayin** | **Magway** | **Mandalay** | **Mon** | **NPT** | **Rakhine** | **Sagaing** | **Shan** | **Tanintharyi** | **Yangon** |
| --- | --- | --- | --- | --- | --- | --- | --- | --- | --- | --- | --- | --- | --- | --- | --- |
| **Undetermined cause 2018/2019 (n)** | 1,289 | 732 | 101 | 557 | 245 | 978 | 1,649 | 907 | 565 | 686 | 501 | 1,874 | 1,527 | 629 | 485 |
| **Total deaths 2018/2019 (n)** | 6,489 | 4,627 | 750 | 3,856 | 1,546 | 7,092 | 8,691 | 6,540 | 5,099 | 3,585 | 2,192 | 12,576 | 8,711 | 4,468 | 3,944 |
| **Undetermined (2018/2019) (%)** | 20% | 16% | 13% | 14% | 16% | 14% | 19% | 14% | 11% | 19% | 23% | 15% | 18% | 14% | 12% |
