# Supplementary material for: Estimating causes of community death of adults in Myanmar from a nationwide population sample: Application of verbal autopsy
Source: PLOS Glob Public Health. 2023 Nov 1;3(11):e0002426. doi: 10.1371/journal.pgph.0002426 (PMC10619871; doi:10.1371/journal.pgph.0002426)
Supplement: S1 Fig — (DOCX) [file pgph.0002426.s007.docx]

**S1 Fig: Age- sex distribution of death, 2018-2019 verbal autopsies in 42 townships and GBD 2019**
