# Supplementary material for: Estimating causes of community death of adults in Myanmar from a nationwide population sample: Application of verbal autopsy
Source: PLOS Glob Public Health. 2023 Nov 1;3(11):e0002426. doi: 10.1371/journal.pgph.0002426 (PMC10619871; doi:10.1371/journal.pgph.0002426)
Supplement: S2 Fig — (PDF) [file pgph.0002426.s008.pdf]

S2 Fig: Age-distribution of top 15 causes - verbal autopsy compared to GBD2019

Stroke

Stroke	# VA Deaths
Male	10,021
Females	8,253
All (Male & Female)	18,274

Males

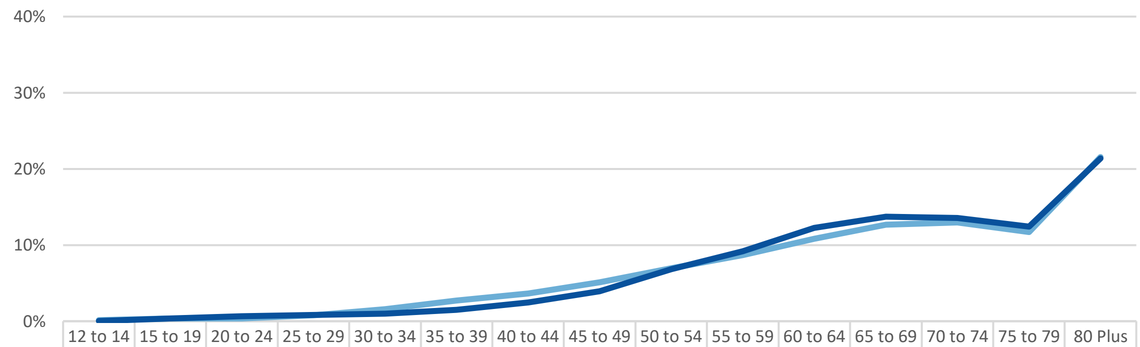

	12 to 14	15 to 19	20 to 24	25 to 29	30 to 34	35 to 39	40 to 44	45 to 49	50 to 54	55 to 59	60 to 64	65 to 69	70 to 74	75 to 79	80 Plus
VA Male	0%	0%	0%	1%	2%	3%	4%	5%	7%	9%	11%	13%	13%	12%	22%
GBD Male	0%	0%	1%	1%	1%	1%	2%	4%	7%	9%	12%	14%	14%	12%	21%

Females

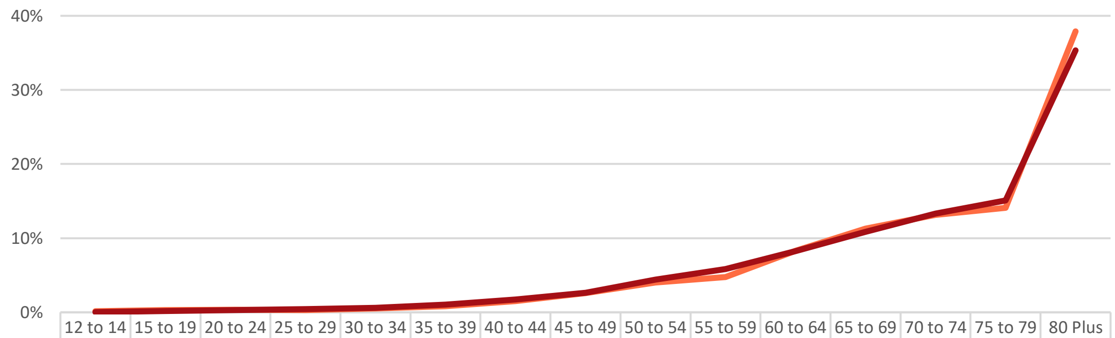

	12 to 14	15 to 19	20 to 24	25 to 29	30 to 34	35 to 39	40 to 44	45 to 49	50 to 54	55 to 59	60 to 64	65 to 69	70 to 74	75 to 79	80 Plus
VA Female	0%	0%	0%	0%	1%	1%	2%	3%	4%	5%	8%	11%	13%	14%	38%
GBD Female	0%	0%	0%	0%	1%	1%	2%	3%	4%	6%	8%	11%	13%	15%	35%

Both (Males and Females)

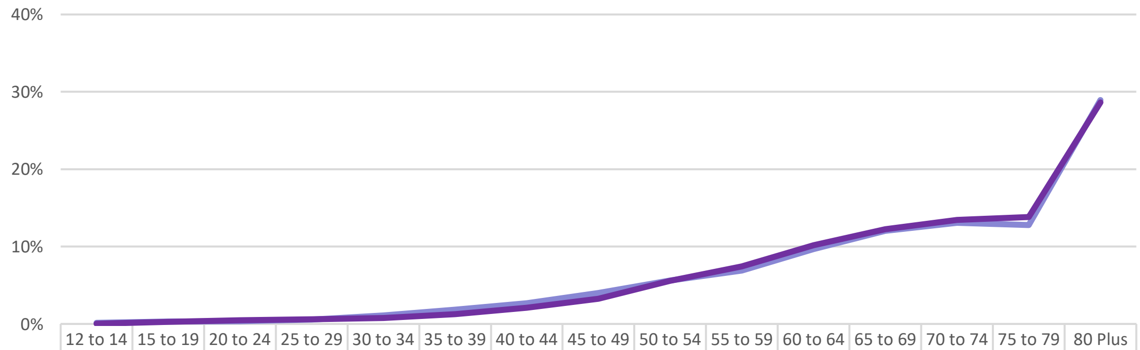

	12 to 14	15 to 19	20 to 24	25 to 29	30 to 34	35 to 39	40 to 44	45 to 49	50 to 54	55 to 59	60 to 64	65 to 69	70 to 74	75 to 79	80 Plus
VA Both	0%	0%	0%	1%	1%	2%	3%	4%	6%	7%	10%	12%	13%	13%	29%
GBD Both	0%	0%	0%	1%	1%	1%	2%	3%	6%	7%	10%	12%	13%	14%	29%

Ischemic Heart Disease

Ischemic Heart Disease	# VA Deaths
Male	3,961
Females	5,158
All (Male & Female)	9,119

Males

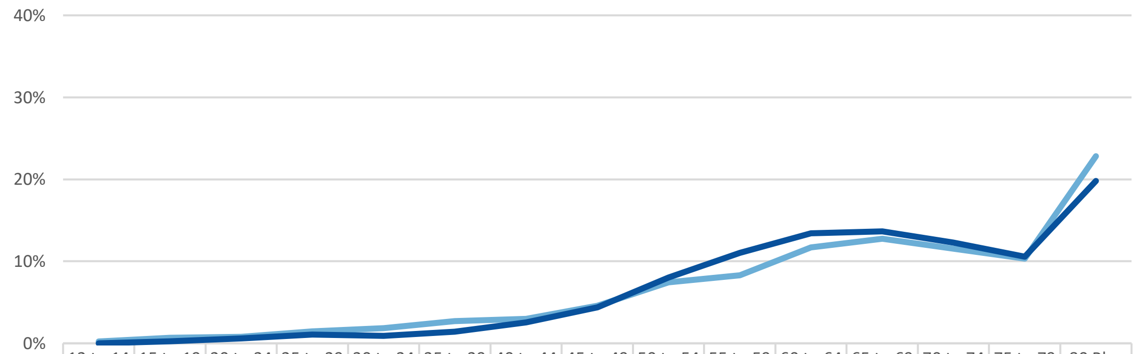

	12 to 14	15 to 19	20 to 24	25 to 29	30 to 34	35 to 39	40 to 44	45 to 49	50 to 54	55 to 59	60 to 64	65 to 69	70 to 74	75 to 79	80 Plus
VA Male	0%	1%	1%	1%	2%	3%	3%	5%	7%	8%	12%	13%	12%	10%	23%
GBD Male	0%	0%	1%	1%	1%	1%	3%	4%	8%	11%	13%	14%	12%	11%	20%

Females

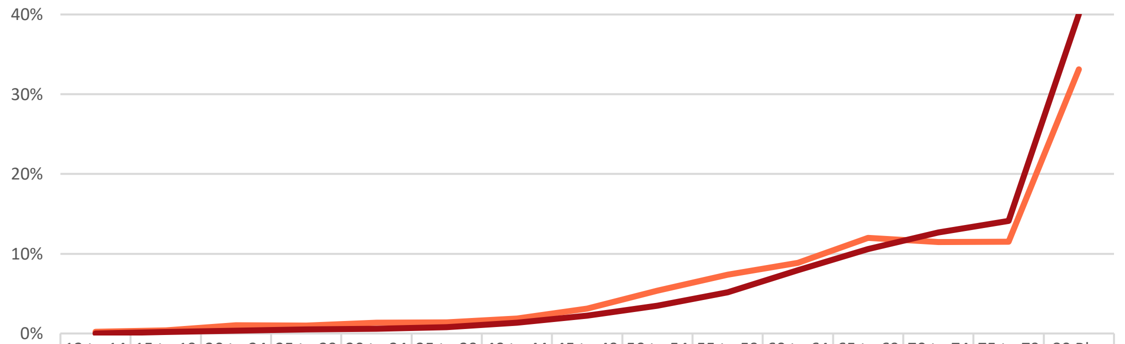

	12 to 14	15 to 19	20 to 24	25 to 29	30 to 34	35 to 39	40 to 44	45 to 49	50 to 54	55 to 59	60 to 64	65 to 69	70 to 74	75 to 79	80 Plus
VA Female	0%	0%	1%	1%	1%	1%	2%	3%	5%	7%	9%	12%	11%	12%	33%
GBD Female	0%	0%	0%	1%	1%	1%	1%	2%	3%	5%	8%	11%	13%	14%	40%

Both (Males and Females)

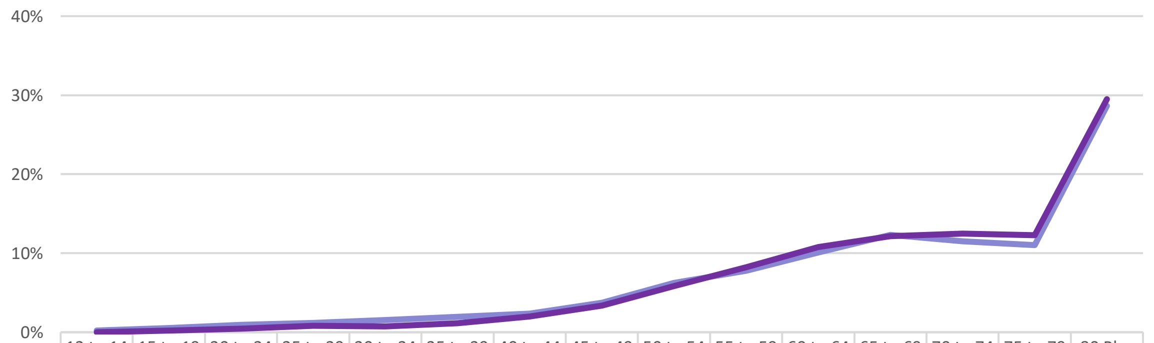

	12 to 14	15 to 19	20 to 24	25 to 29	30 to 34	35 to 39	40 to 44	45 to 49	50 to 54	55 to 59	60 to 64	65 to 69	70 to 74	75 to 79	80 Plus
VA Both	0%	1%	1%	1%	2%	2%	2%	4%	6%	8%	10%	12%	12%	11%	29%
GBD Both	0%	0%	0%	1%	1%	1%	2%	3%	6%	8%	11%	12%	12%	12%	29%

Chronic Respiratory

Chronic Respiratory	# VA Deaths
Male	3,913
Females	3,814
All (Male & Female)	7,727

Males

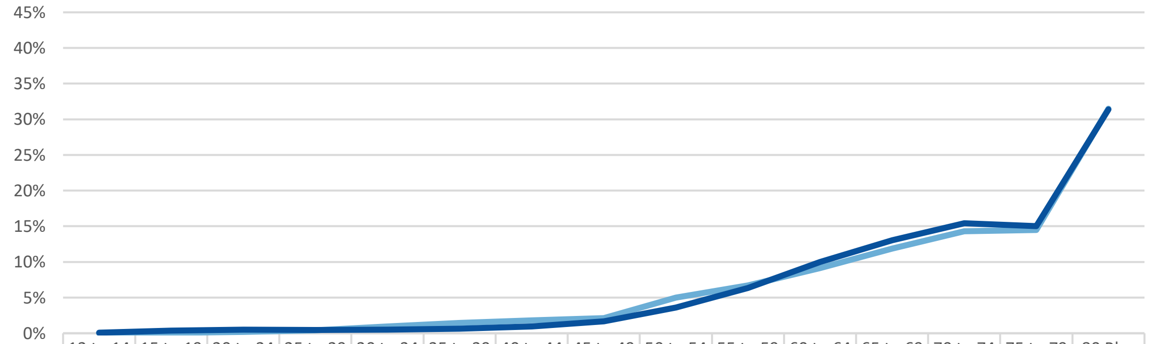

	12 to 14	15 to 19	20 to 24	25 to 29	30 to 34	35 to 39	40 to 44	45 to 49	50 to 54	55 to 59	60 to 64	65 to 69	70 to 74	75 to 79	80 Plus
VA Male	0%	0%	0%	0%	1%	1%	2%	2%	5%	7%	9%	12%	14%	14%	31%
GBD Male	0%	0%	0%	0%	1%	1%	1%	2%	4%	6%	10%	13%	15%	15%	31%

Females

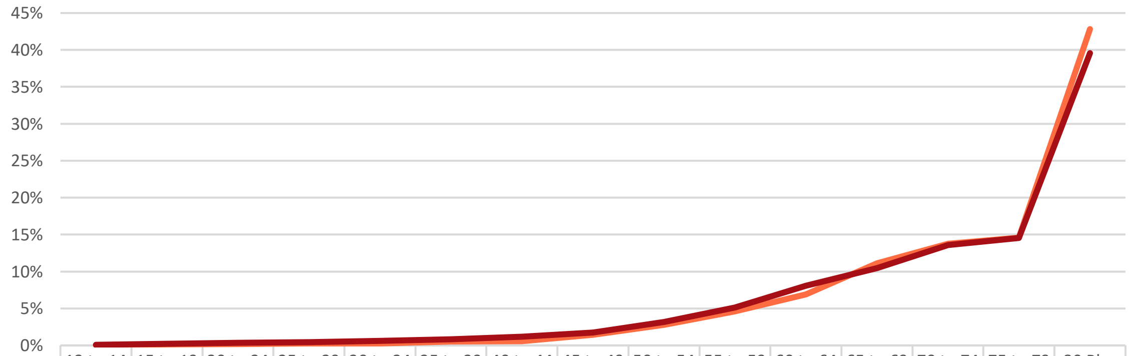

	12 to 14	15 to 19	20 to 24	25 to 29	30 to 34	35 to 39	40 to 44	45 to 49	50 to 54	55 to 59	60 to 64	65 to 69	70 to 74	75 to 79	80 Plus
VA Female	0%	0%	0%	0%	0%	1%	1%	1%	3%	5%	7%	11%	14%	15%	43%
GBD Female	0%	0%	0%	0%	1%	1%	1%	2%	3%	5%	8%	10%	14%	15%	40%

Both (Males and Females)

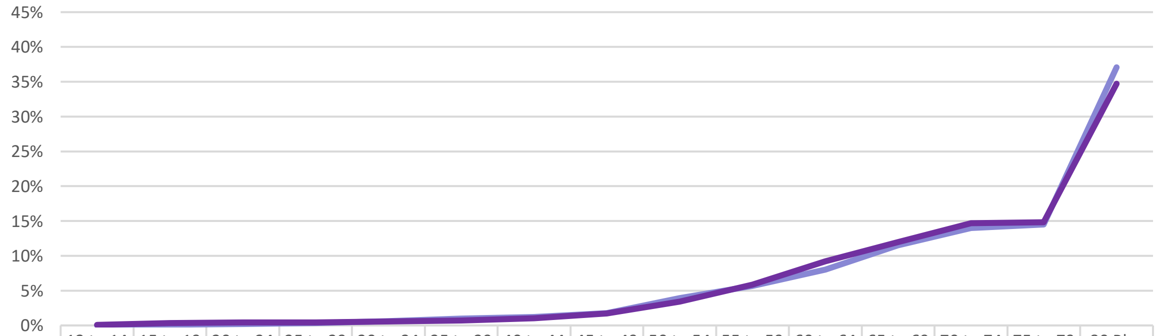

	12 to 14	15 to 19	20 to 24	25 to 29	30 to 34	35 to 39	40 to 44	45 to 49	50 to 54	55 to 59	60 to 64	65 to 69	70 to 74	75 to 79	80 Plus
VA Both	0%	0%	0%	0%	1%	1%	1%	2%	4%	6%	8%	11%	14%	15%	37%
GBD Both	0%	0%	0%	0%	1%	1%	1%	2%	3%	6%	9%	12%	15%	15%	35%

Cirrhosis

Cirrhosis	# VA Deaths
Male	5,736
Females	923
All (Male & Female)	6,659

Males

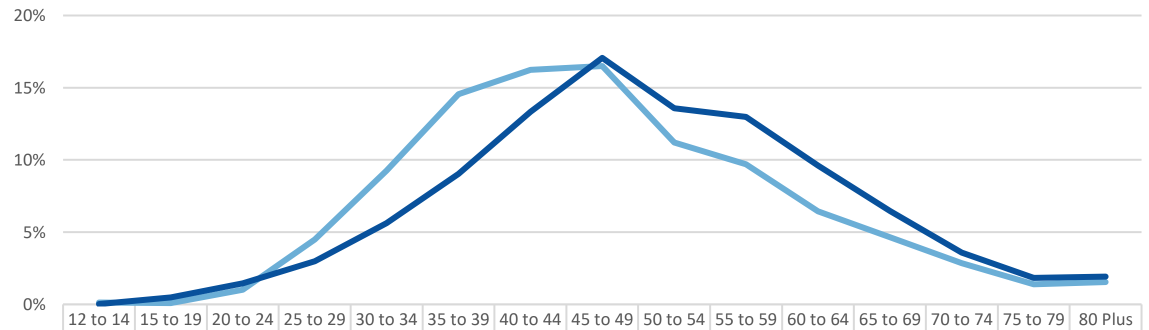

	12 to 14	15 to 19	20 to 24	25 to 29	30 to 34	35 to 39	40 to 44	45 to 49	50 to 54	55 to 59	60 to 64	65 to 69	70 to 74	75 to 79	80 Plus
VA Male	0%	0%	1%	5%	9%	15%	16%	17%	11%	10%	6%	5%	3%	1%	2%
GBD Male	0%	0%	1%	3%	6%	9%	13%	17%	14%	13%	10%	6%	4%	2%	2%

Females

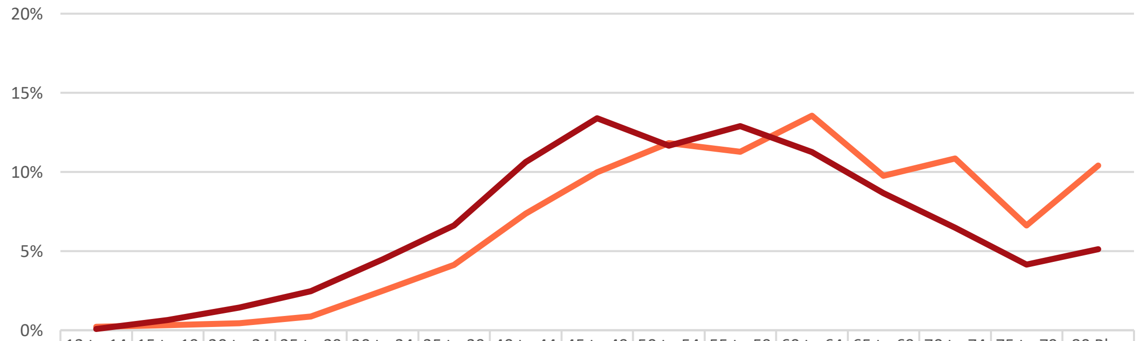

	12 to 14	15 to 19	20 to 24	25 to 29	30 to 34	35 to 39	40 to 44	45 to 49	50 to 54	55 to 59	60 to 64	65 to 69	70 to 74	75 to 79	80 Plus
VA Female	0%	0%	0%	1%	2%	4%	7%	10%	12%	11%	14%	10%	11%	7%	10%
GBD Female	0%	1%	1%	2%	4%	7%	11%	13%	12%	13%	11%	9%	6%	4%	5%

Both (Males and Females)

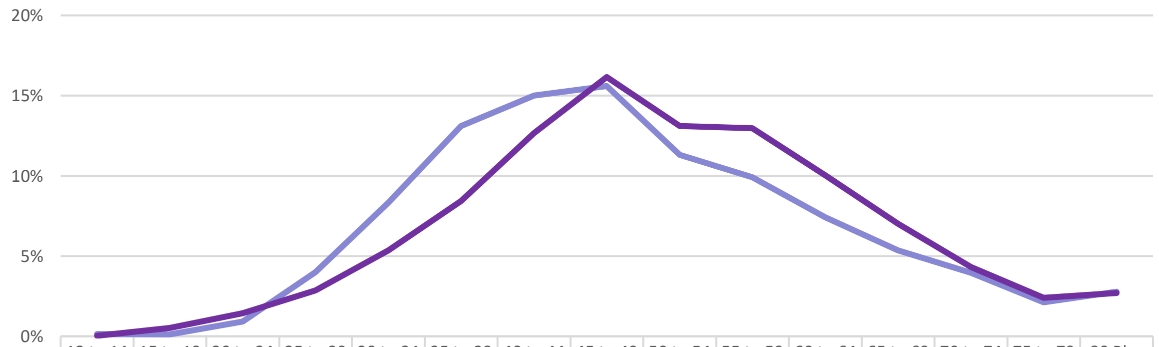

	12 to 14	15 to 19	20 to 24	25 to 29	30 to 34	35 to 39	40 to 44	45 to 49	50 to 54	55 to 59	60 to 64	65 to 69	70 to 74	75 to 79	80 Plus
VA Both	0%	0%	1%	4%	8%	13%	15%	16%	11%	10%	7%	5%	4%	2%	3%
GBD Both	0%	1%	1%	3%	5%	8%	13%	16%	13%	13%	10%	7%	4%	2%	3%

Diabetes

Diabetes	# VA Deaths
Male	2,183
Females	2,611
All (Male & Female)	4,794

Males

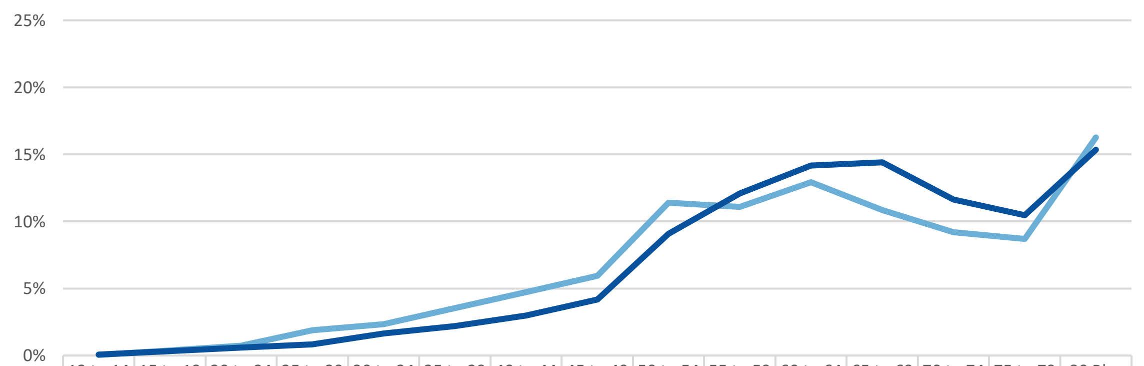

	12 to 14	15 to 19	20 to 24	25 to 29	30 to 34	35 to 39	40 to 44	45 to 49	50 to 54	55 to 59	60 to 64	65 to 69	70 to 74	75 to 79	80 Plus
VA Male	0%	0%	1%	2%	2%	4%	5%	6%	11%	11%	13%	11%	9%	9%	16%
GBD Male	0%	0%	1%	1%	2%	2%	3%	4%	9%	12%	14%	14%	12%	10%	15%

Females

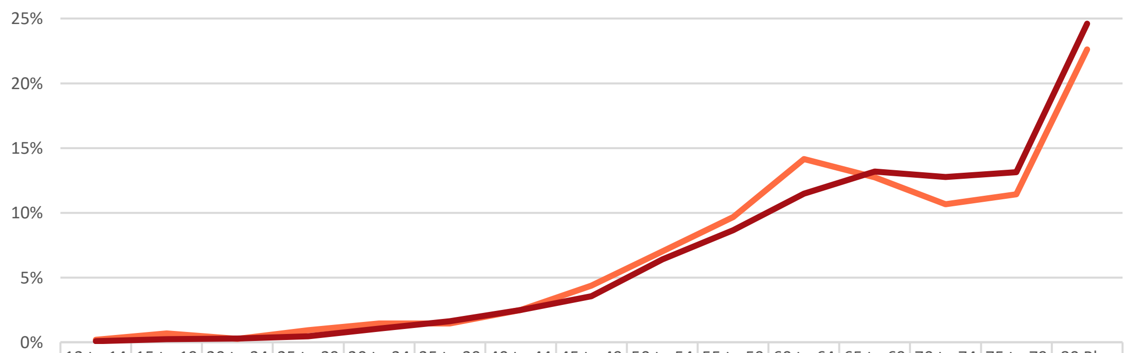

	12 to 14	15 to 19	20 to 24	25 to 29	30 to 34	35 to 39	40 to 44	45 to 49	50 to 54	55 to 59	60 to 64	65 to 69	70 to 74	75 to 79	80 Plus
VA Female	0%	1%	0%	1%	1%	1%	2%	4%	7%	10%	14%	13%	11%	11%	23%
GBD Female	0%	0%	0%	0%	1%	2%	2%	4%	6%	9%	11%	13%	13%	13%	25%

Both (Males and Females)

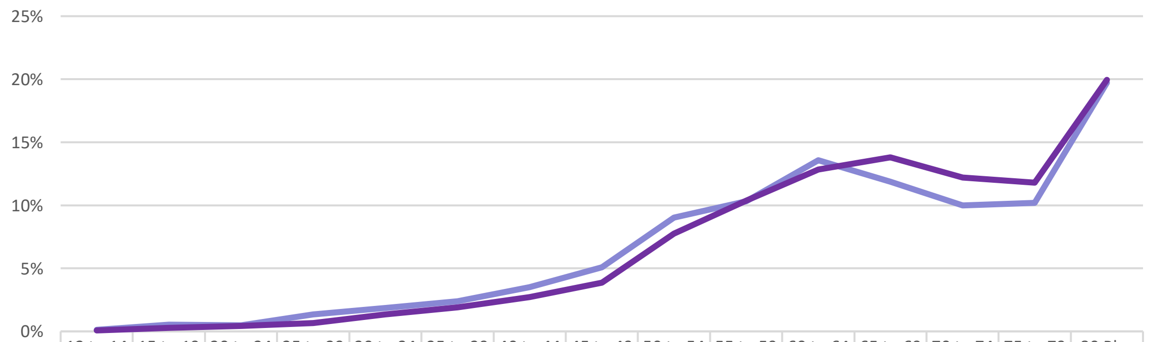

	12 to 14	15 to 19	20 to 24	25 to 29	30 to 34	35 to 39	40 to 44	45 to 49	50 to 54	55 to 59	60 to 64	65 to 69	70 to 74	75 to 79	80 Plus
VA Both	0%	1%	0%	1%	2%	2%	4%	5%	9%	10%	14%	12%	10%	10%	20%
GBD Both	0%	0%	0%	1%	1%	2%	3%	4%	8%	10%	13%	14%	12%	12%	20%

Other non-communicable diseases

Other Non-communicable Diseases	# VA Deaths
Male	1,901
Females	967
All (Male & Female)	2,868

Males

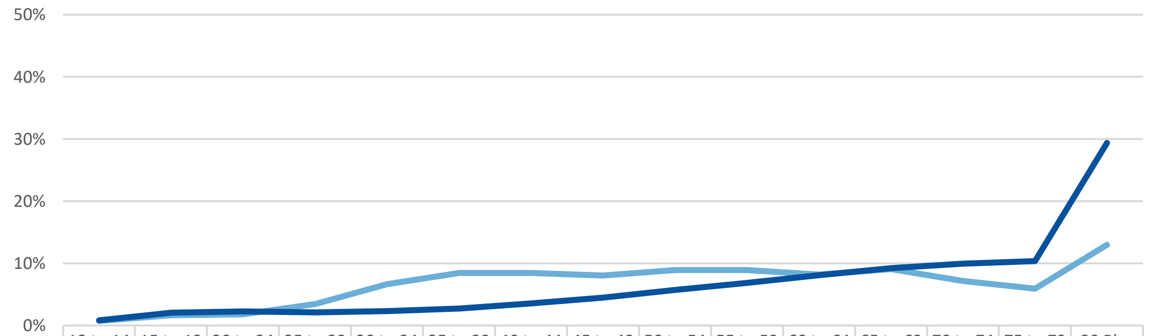

	12 to 14	15 to 19	20 to 24	25 to 29	30 to 34	35 to 39	40 to 44	45 to 49	50 to 54	55 to 59	60 to 64	65 to 69	70 to 74	75 to 79	80 Plus
VA Male	1%	2%	2%	3%	7%	8%	8%	8%	9%	9%	8%	9%	7%	6%	13%
GBD Male	1%	2%	2%	2%	2%	3%	4%	4%	6%	7%	8%	9%	10%	10%	29%

Females

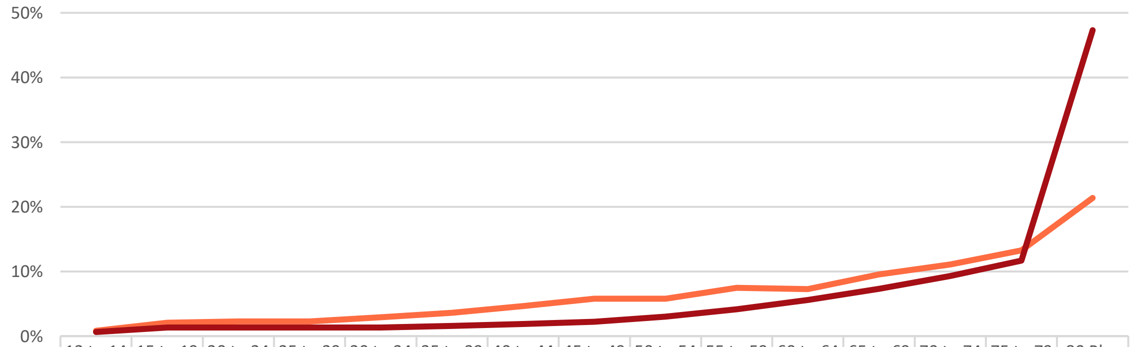

	12 to 14	15 to 19	20 to 24	25 to 29	30 to 34	35 to 39	40 to 44	45 to 49	50 to 54	55 to 59	60 to 64	65 to 69	70 to 74	75 to 79	80 Plus
VA Female	1%	2%	2%	2%	3%	4%	5%	6%	6%	7%	7%	10%	11%	13%	21%
GBD Female	1%	1%	1%	1%	1%	2%	2%	2%	3%	4%	6%	7%	9%	12%	47%

Both (Males and Females)

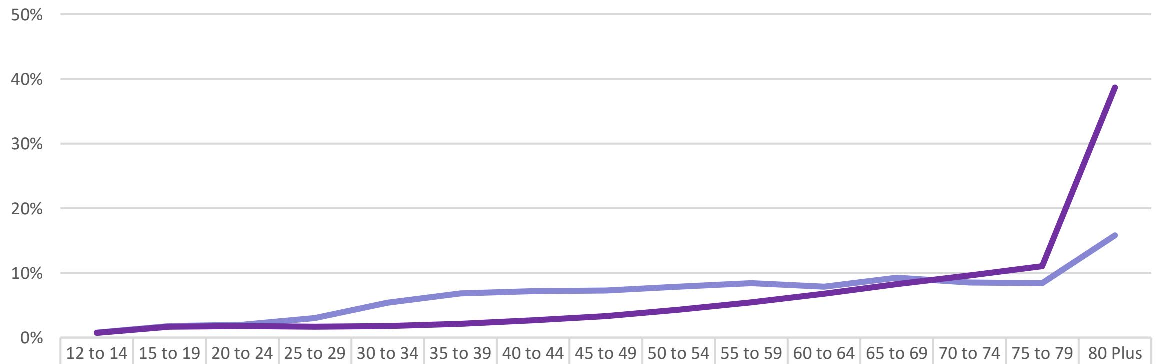

	12 to 14	15 to 19	20 to 24	25 to 29	30 to 34	35 to 39	40 to 44	45 to 49	50 to 54	55 to 59	60 to 64	65 to 69	70 to 74	75 to 79	80 Plus
VA Both	1%	2%	2%	3%	5%	7%	7%	7%	8%	8%	8%	9%	8%	8%	16%
GBD Both	1%	2%	2%	2%	2%	2%	3%	3%	4%	5%	7%	8%	10%	11%	39%

Pneumonia

**Pneumonia**	**# VA Deaths**
Male	1,545
Females	1,069
All (Male & Female)	2,614

Males

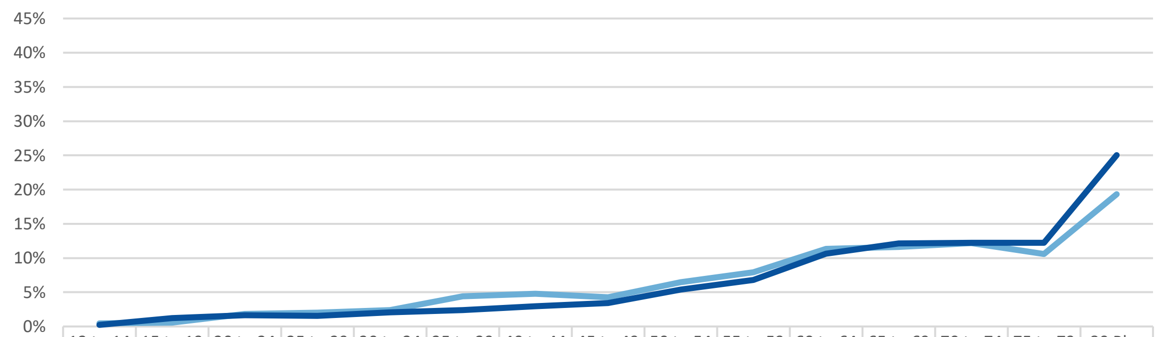

	12 to 14	15 to 19	20 to 24	25 to 29	30 to 34	35 to 39	40 to 44	45 to 49	50 to 54	55 to 59	60 to 64	65 to 69	70 to 74	75 to 79	80 Plus
VA Male	0%	1%	2%	2%	2%	4%	5%	4%	6%	8%	11%	12%	12%	11%	19%
GBD Male	0%	1%	2%	2%	2%	2%	3%	3%	5%	7%	11%	12%	12%	12%	25%

Females

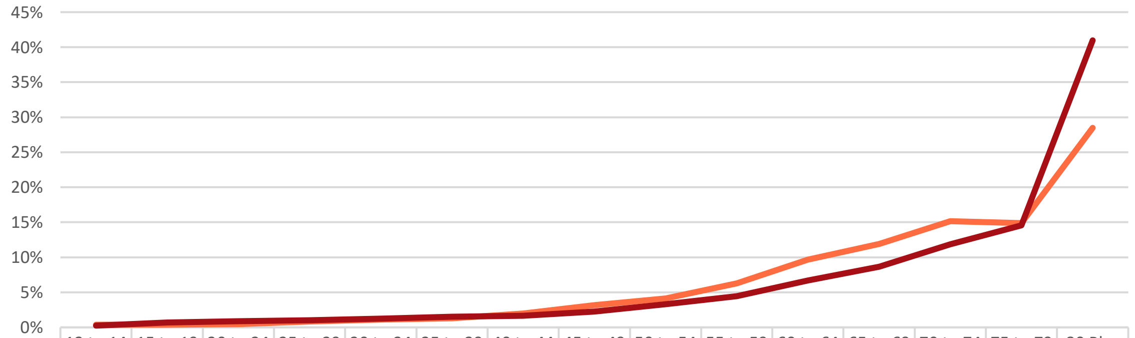

	12 to 14	15 to 19	20 to 24	25 to 29	30 to 34	35 to 39	40 to 44	45 to 49	50 to 54	55 to 59	60 to 64	65 to 69	70 to 74	75 to 79	80 Plus
VA Female	0%	0%	0%	1%	1%	1%	2%	3%	4%	6%	10%	12%	15%	15%	28%
GBD Female	0%	1%	1%	1%	1%	1%	2%	2%	3%	4%	7%	9%	12%	15%	41%

Both (Males and Females)

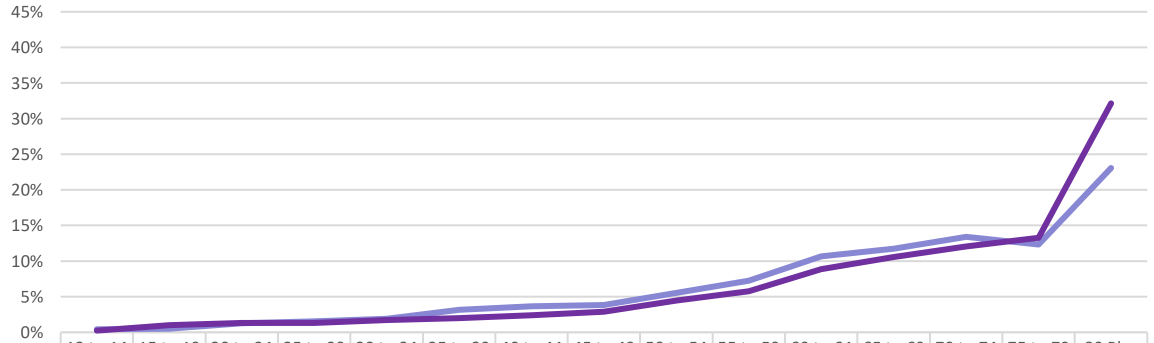

	12 to 14	15 to 19	20 to 24	25 to 29	30 to 34	35 to 39	40 to 44	45 to 49	50 to 54	55 to 59	60 to 64	65 to 69	70 to 74	75 to 79	80 Plus
VA Both	0%	0%	1%	2%	2%	3%	4%	4%	6%	7%	11%	12%	13%	12%	23%
GBD Both	0%	1%	1%	1%	2%	2%	2%	3%	4%	6%	9%	11%	12%	13%	32%

Chronic Kidney Disease

**Chronic Kidney Disease**	**# VA Deaths**
Male	1,098
Females	874
All (Male & Female)	1,972

Males

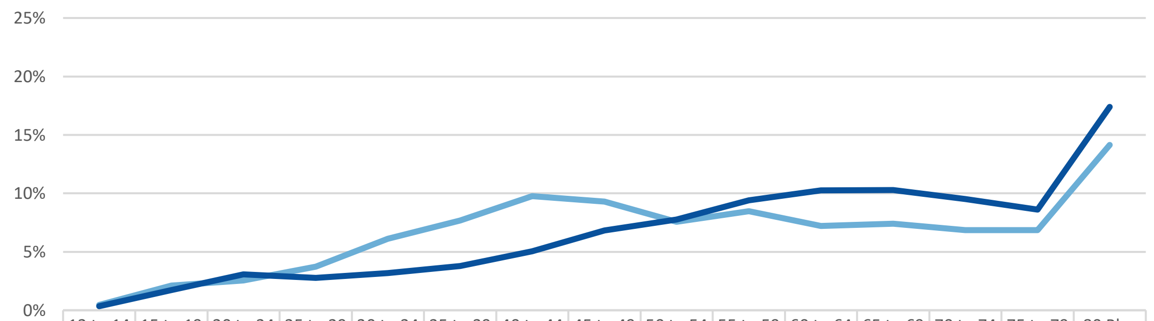

	12 to 14	15 to 19	20 to 24	25 to 29	30 to 34	35 to 39	40 to 44	45 to 49	50 to 54	55 to 59	60 to 64	65 to 69	70 to 74	75 to 79	80 Plus
VA Male	0%	2%	3%	4%	6%	8%	10%	9%	8%	8%	7%	7%	7%	7%	14%
GBD Male	0%	2%	3%	3%	3%	4%	5%	7%	8%	9%	10%	10%	10%	9%	17%

Females

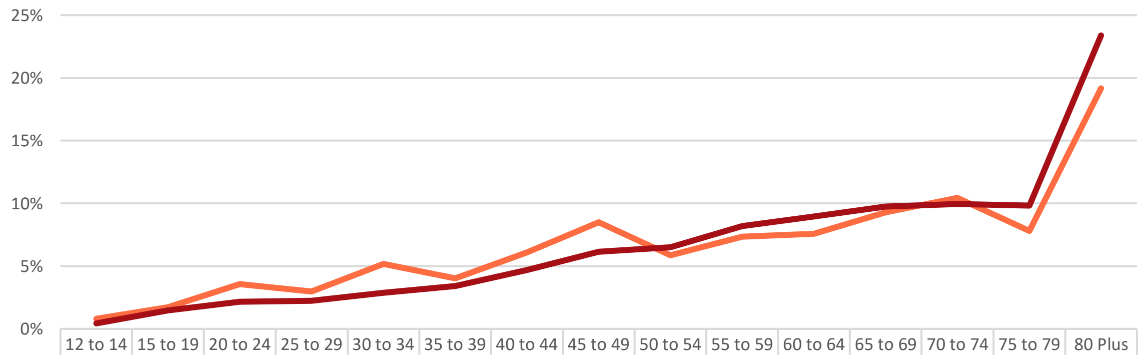

	12 to 14	15 to 19	20 to 24	25 to 29	30 to 34	35 to 39	40 to 44	45 to 49	50 to 54	55 to 59	60 to 64	65 to 69	70 to 74	75 to 79	80 Plus
VA Female	1%	2%	4%	3%	5%	4%	6%	8%	6%	7%	8%	9%	10%	8%	19%
GBD Female	0%	1%	2%	2%	3%	3%	5%	6%	6%	8%	9%	10%	10%	10%	23%

Both (Males and Females)

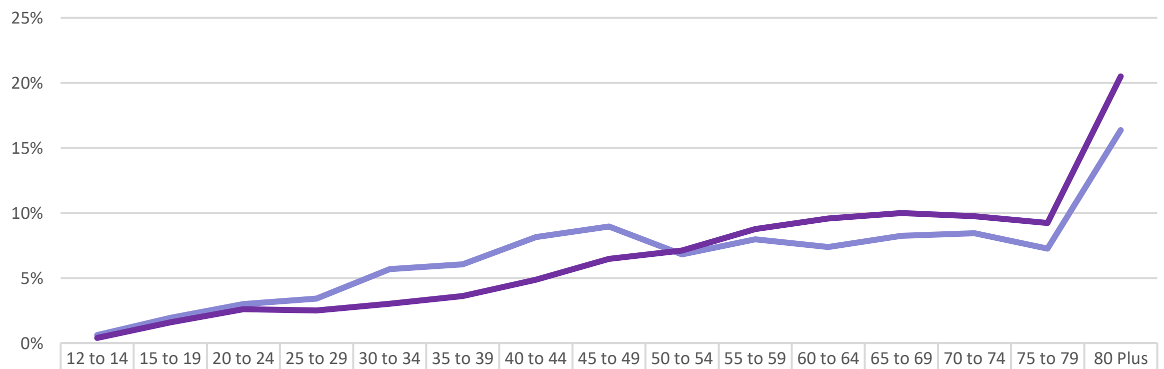

	12 to 14	15 to 19	20 to 24	25 to 29	30 to 34	35 to 39	40 to 44	45 to 49	50 to 54	55 to 59	60 to 64	65 to 69	70 to 74	75 to 79	80 Plus
VA Both	1%	2%	3%	3%	6%	6%	8%	9%	7%	8%	7%	8%	8%	7%	16%
GBD Both	0%	2%	3%	2%	3%	4%	5%	6%	7%	9%	10%	10%	10%	9%	21%

Tuberculosis

TB	# VA Deaths
Male	1,190
Females	481
All (Male & Female)	1,671

Males

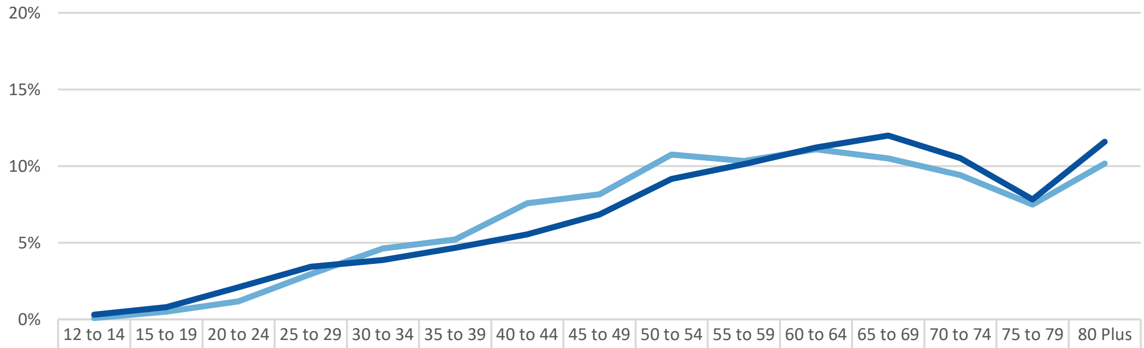

	12 to 14	15 to 19	20 to 24	25 to 29	30 to 34	35 to 39	40 to 44	45 to 49	50 to 54	55 to 59	60 to 64	65 to 69	70 to 74	75 to 79	80 Plus
VA Male	0%	1%	1%	3%	5%	5%	8%	8%	11%	10%	11%	11%	9%	7%	10%
GBD Male	0%	1%	2%	3%	4%	5%	6%	7%	9%	10%	11%	12%	11%	8%	12%

Females

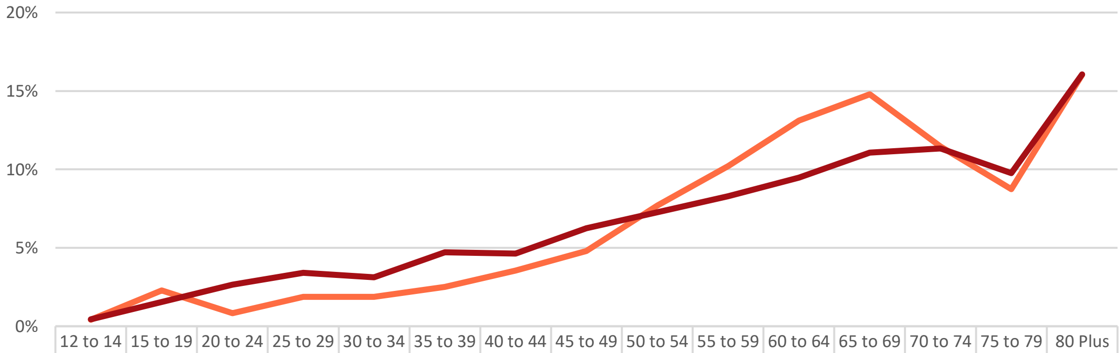

	12 to 14	15 to 19	20 to 24	25 to 29	30 to 34	35 to 39	40 to 44	45 to 49	50 to 54	55 to 59	60 to 64	65 to 69	70 to 74	75 to 79	80 Plus
VA Female	0%	2%	1%	2%	2%	2%	4%	5%	8%	10%	13%	15%	11%	9%	16%
GBD Female	0%	2%	3%	3%	3%	5%	5%	6%	7%	8%	9%	11%	11%	10%	16%

Both (Males and Females)

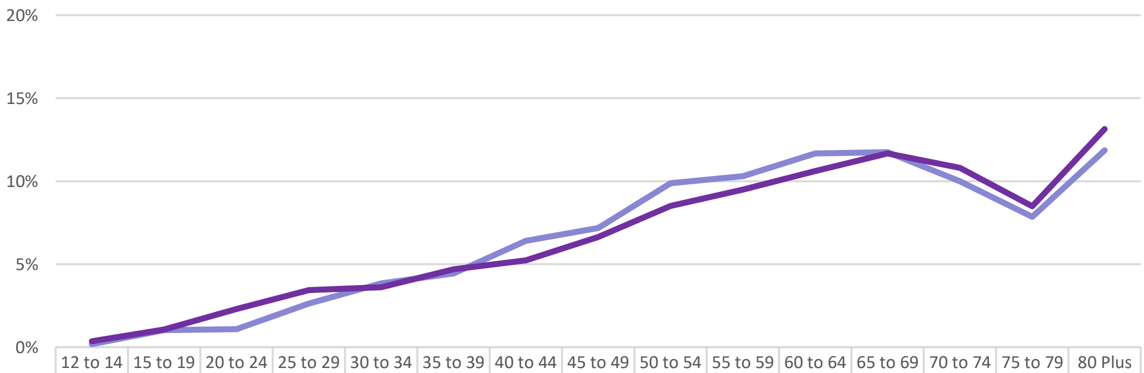

	12 to 14	15 to 19	20 to 24	25 to 29	30 to 34	35 to 39	40 to 44	45 to 49	50 to 54	55 to 59	60 to 64	65 to 69	70 to 74	75 to 79	80 Plus
VA Both	0%	1%	1%	3%	4%	4%	6%	7%	10%	10%	12%	12%	10%	8%	12%
GBD Both	0%	1%	2%	3%	4%	5%	5%	7%	9%	9%	11%	12%	11%	8%	13%

Leukemia/Lymphoma

**Leukemia/Lymphomas**	**# VA Deaths**
Male	656
Females	508
All (Male & Female)	1,164

Males

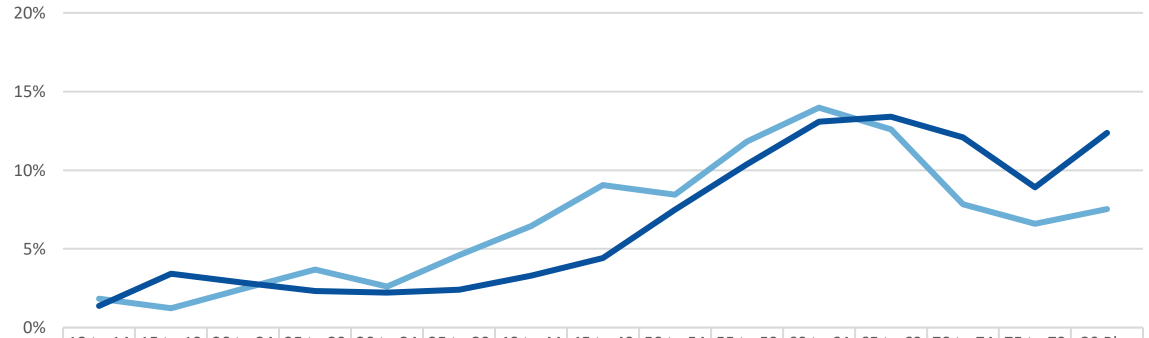

	12 to 14	15 to 19	20 to 24	25 to 29	30 to 34	35 to 39	40 to 44	45 to 49	50 to 54	55 to 59	60 to 64	65 to 69	70 to 74	75 to 79	80 Plus
VA Male	2%	1%	2%	4%	3%	5%	6%	9%	8%	12%	14%	13%	8%	7%	8%
GBD Male	1%	3%	3%	2%	2%	2%	3%	4%	7%	10%	13%	13%	12%	9%	12%

Females

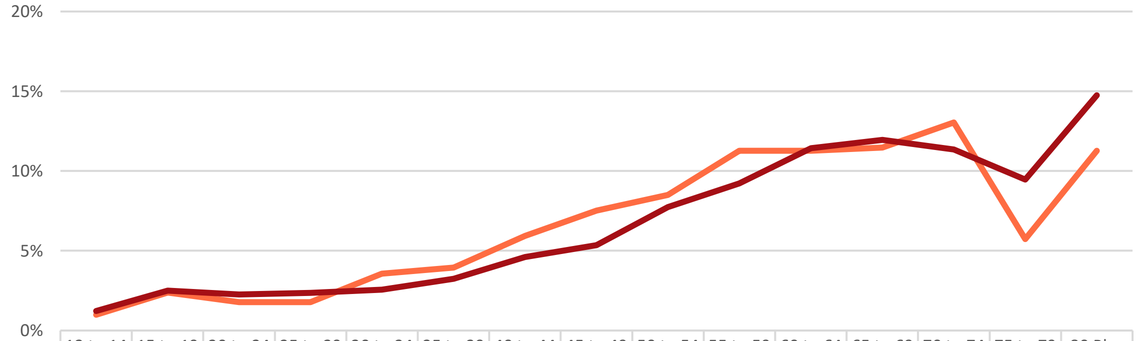

	12 to 14	15 to 19	20 to 24	25 to 29	30 to 34	35 to 39	40 to 44	45 to 49	50 to 54	55 to 59	60 to 64	65 to 69	70 to 74	75 to 79	80 Plus
VA Female	1%	2%	2%	2%	4%	4%	6%	8%	8%	11%	11%	11%	13%	6%	11%
GBD Female	1%	3%	2%	2%	3%	3%	5%	5%	8%	9%	11%	12%	11%	9%	15%

Both (Males and Females)

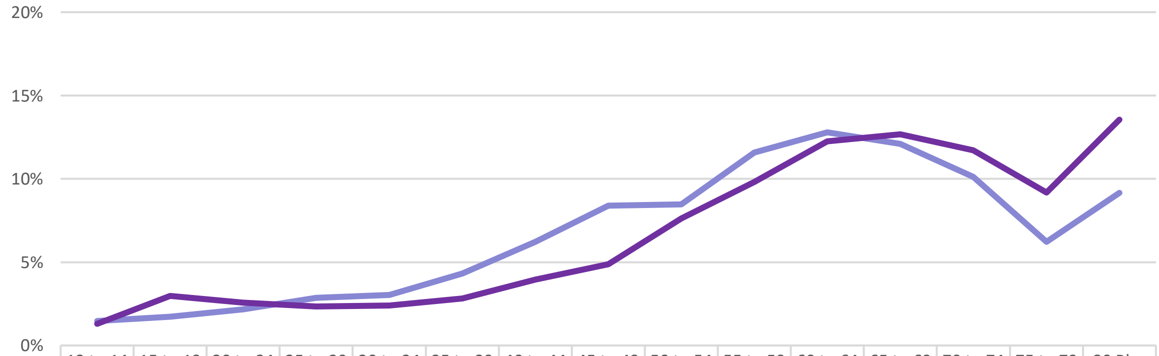

	12 to 14	15 to 19	20 to 24	25 to 29	30 to 34	35 to 39	40 to 44	45 to 49	50 to 54	55 to 59	60 to 64	65 to 69	70 to 74	75 to 79	80 Plus
VA Both	1%	2%	2%	3%	3%	4%	6%	8%	8%	12%	13%	12%	10%	6%	9%
GBD Both	1%	3%	3%	2%	2%	3%	4%	5%	8%	10%	12%	13%	12%	9%	14%

AIDS

AIDS	# VA Deaths
Male	661
Females	307
All (Male & Female)	968

Males

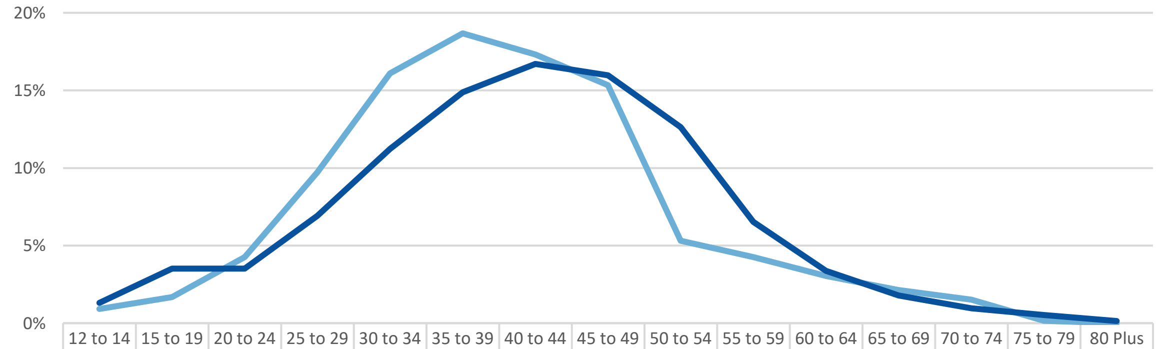

	12 to 14	15 to 19	20 to 24	25 to 29	30 to 34	35 to 39	40 to 44	45 to 49	50 to 54	55 to 59	60 to 64	65 to 69	70 to 74	75 to 79	80 Plus
VA Male	1%	2%	4%	10%	16%	19%	17%	15%	5%	4%	3%	2%	2%	0%	0%
GBD Male	1%	4%	4%	7%	11%	15%	17%	16%	13%	7%	3%	2%	1%	1%	0%

Females

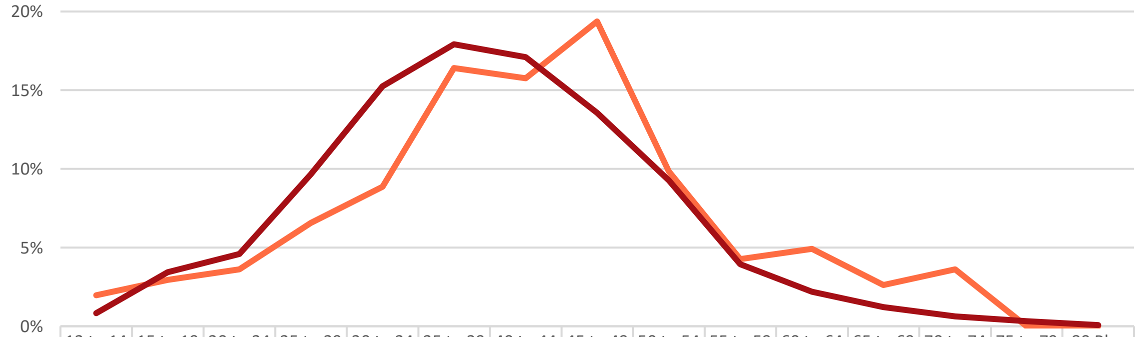

	12 to 14	15 to 19	20 to 24	25 to 29	30 to 34	35 to 39	40 to 44	45 to 49	50 to 54	55 to 59	60 to 64	65 to 69	70 to 74	75 to 79	80 Plus
VA Female	2%	3%	4%	7%	9%	16%	16%	19%	10%	4%	5%	3%	4%	0%	0%
GBD Female	1%	3%	5%	10%	15%	18%	17%	14%	9%	4%	2%	1%	1%	0%	0%

Both (Males and Females)

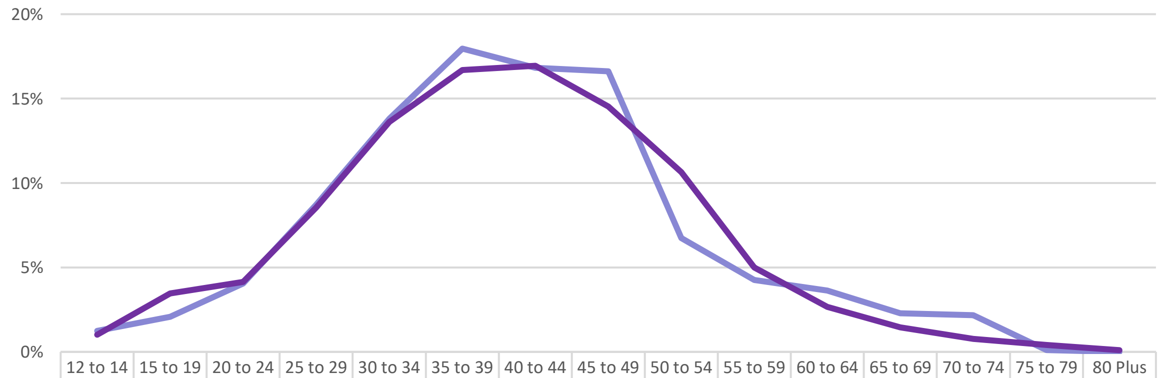

	12 to 14	15 to 19	20 to 24	25 to 29	30 to 34	35 to 39	40 to 44	45 to 49	50 to 54	55 to 59	60 to 64	65 to 69	70 to 74	75 to 79	80 Plus
VA Both	1%	2%	4%	9%	14%	18%	17%	17%	7%	4%	4%	2%	2%	0%	0%
GBD Both	1%	3%	4%	9%	14%	17%	17%	15%	11%	5%	3%	1%	1%	0%	0%

Diarrhoa/Dysentery

Diarrhea/Dysentery	# VA Deaths
Male	215
Females	224
All (Male & Female)	439

Males

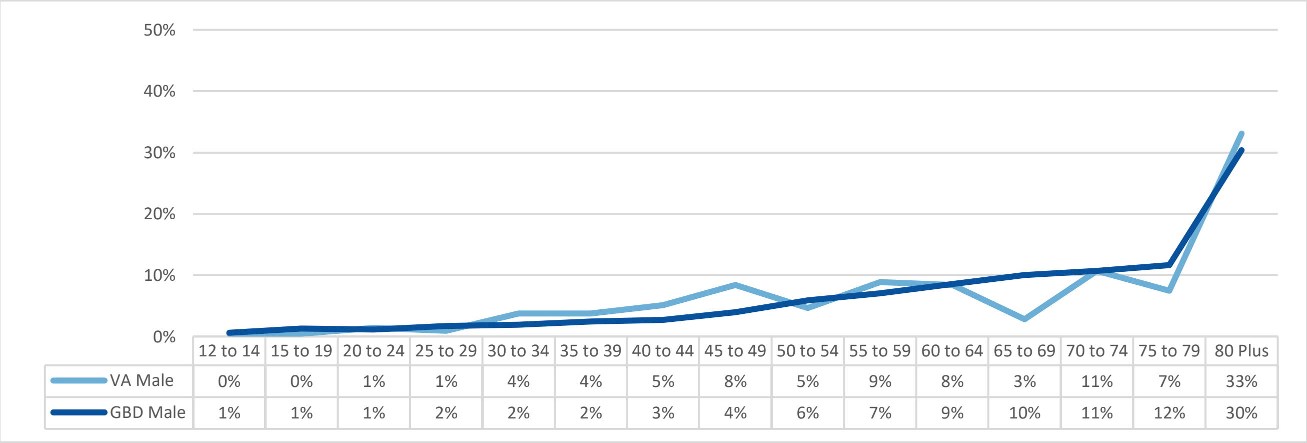

Females

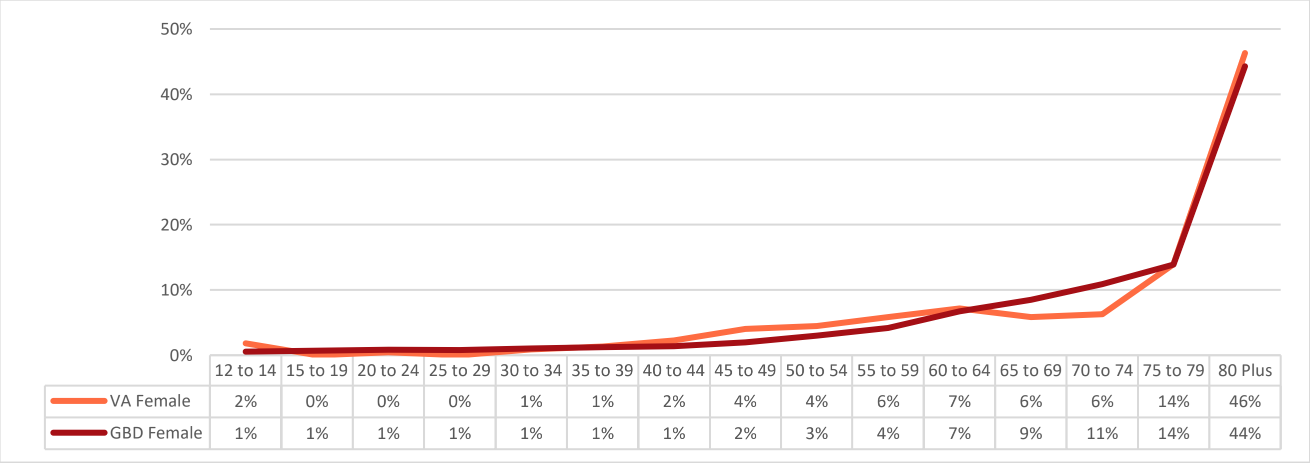

Both (Males and Females)

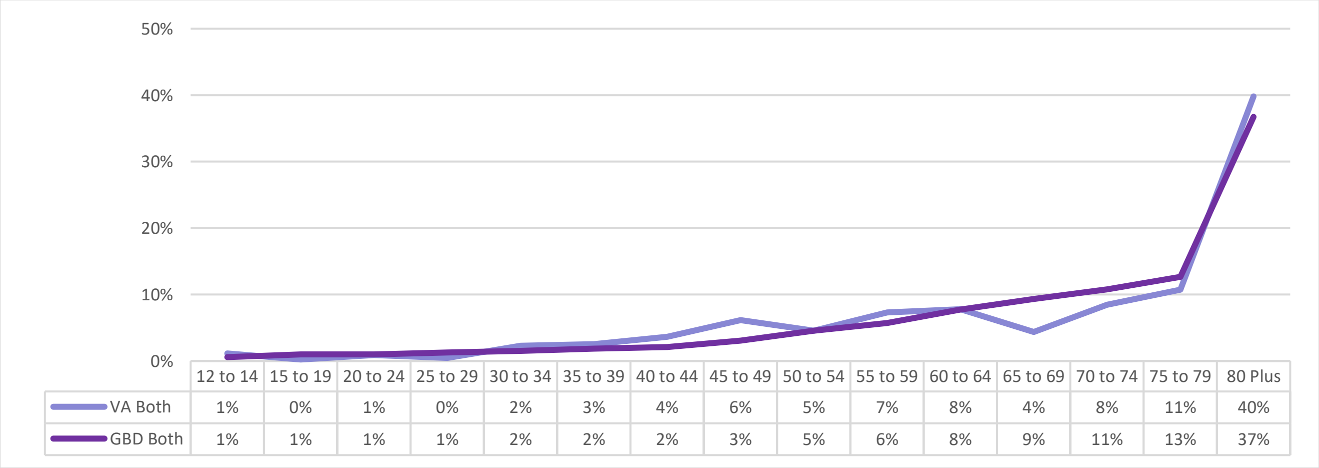

Lung cancer

Lung Cancer	# VA Deaths
Male	619
Females	297
All (Male & Female)	916

Males

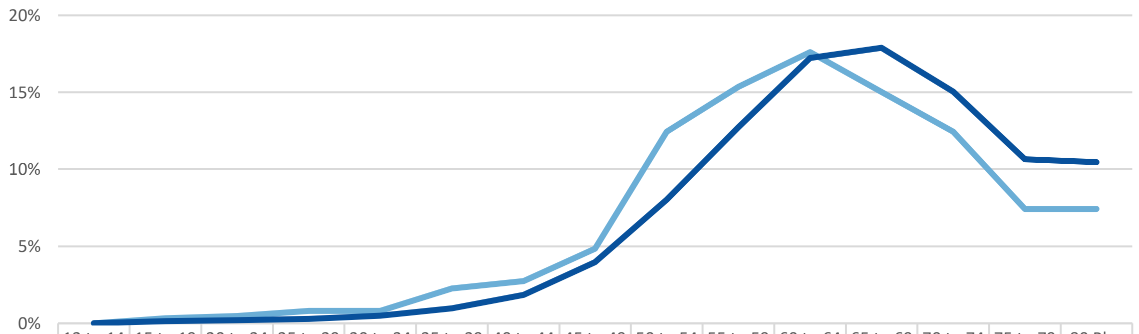

	12 to 14	15 to 19	20 to 24	25 to 29	30 to 34	35 to 39	40 to 44	45 to 49	50 to 54	55 to 59	60 to 64	65 to 69	70 to 74	75 to 79	80 Plus
VA Male	0%	0%	0%	1%	1%	2%	3%	5%	12%	15%	18%	15%	12%	7%	7%
GBD Male	0%	0%	0%	0%	0%	1%	2%	4%	8%	13%	17%	18%	15%	11%	10%

Females

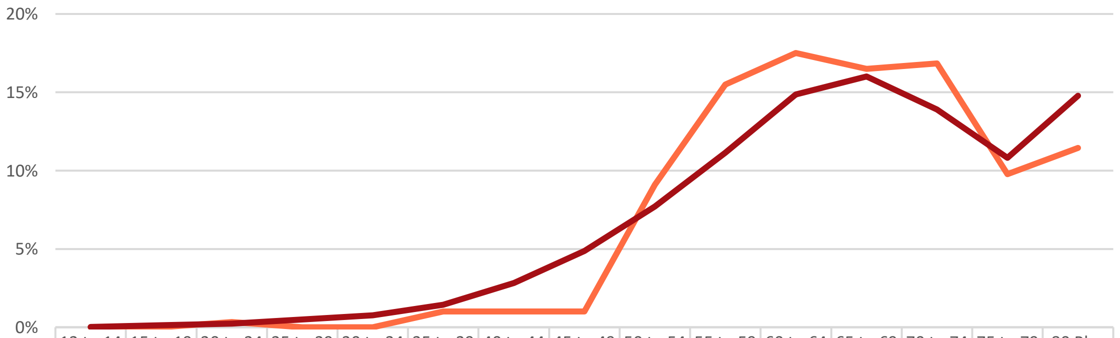

	12 to 14	15 to 19	20 to 24	25 to 29	30 to 34	35 to 39	40 to 44	45 to 49	50 to 54	55 to 59	60 to 64	65 to 69	70 to 74	75 to 79	80 Plus
VA Female	0%	0%	0%	0%	0%	1%	1%	1%	9%	15%	18%	16%	17%	10%	11%
GBD Female	0%	0%	0%	1%	1%	1%	3%	5%	8%	11%	15%	16%	14%	11%	15%

Both (Males and Females)

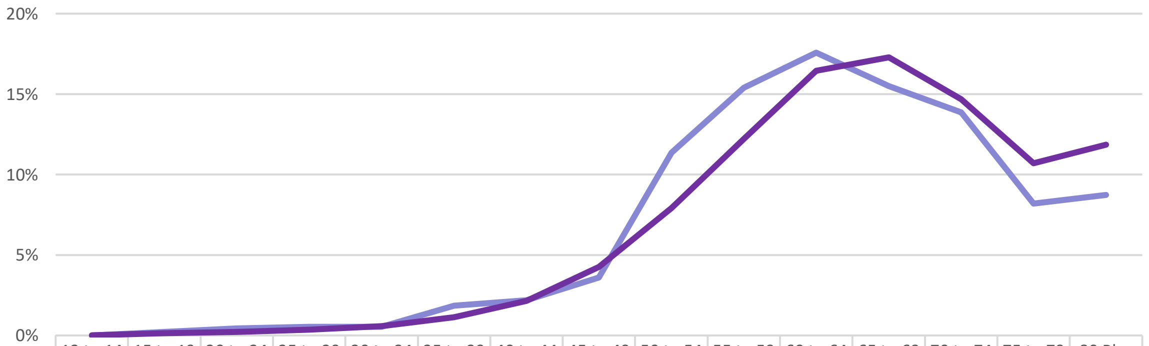

	12 to 14	15 to 19	20 to 24	25 to 29	30 to 34	35 to 39	40 to 44	45 to 49	50 to 54	55 to 59	60 to 64	65 to 69	70 to 74	75 to 79	80 Plus
VA Both	0%	0%	0%	1%	1%	2%	2%	4%	11%	15%	18%	16%	14%	8%	9%
GBD Both	0%	0%	0%	0%	1%	1%	2%	4%	8%	12%	16%	17%	15%	11%	12%

Esophageal Cancer

Esophageal Cancer	# VA Deaths
Male	634
Females	420
All (Male & Female)	1,054

Males

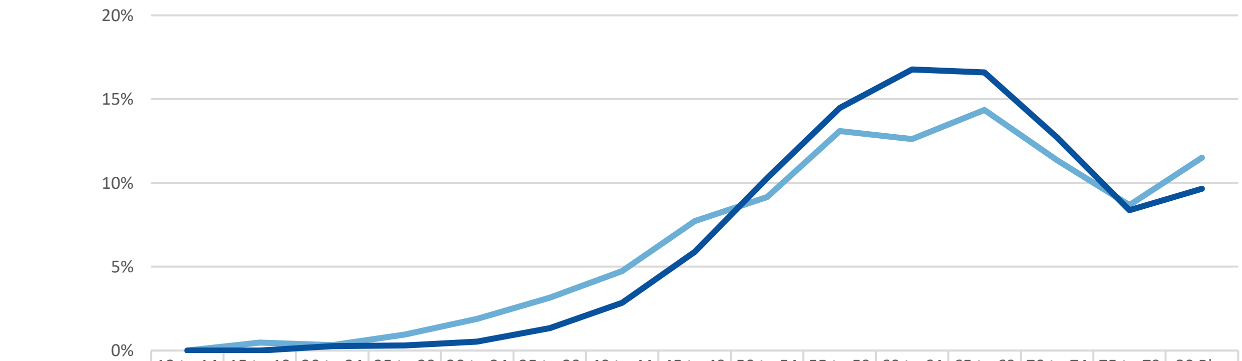

	12 to 14	15 to 19	20 to 24	25 to 29	30 to 34	35 to 39	40 to 44	45 to 49	50 to 54	55 to 59	60 to 64	65 to 69	70 to 74	75 to 79	80 Plus
VA Male	0%	0%	0%	1%	2%	3%	5%	8%	9%	13%	13%	14%	11%	9%	12%
GBD Male	0%	0%	0%	0%	1%	1%	3%	6%	10%	14%	17%	17%	13%	8%	10%

Females

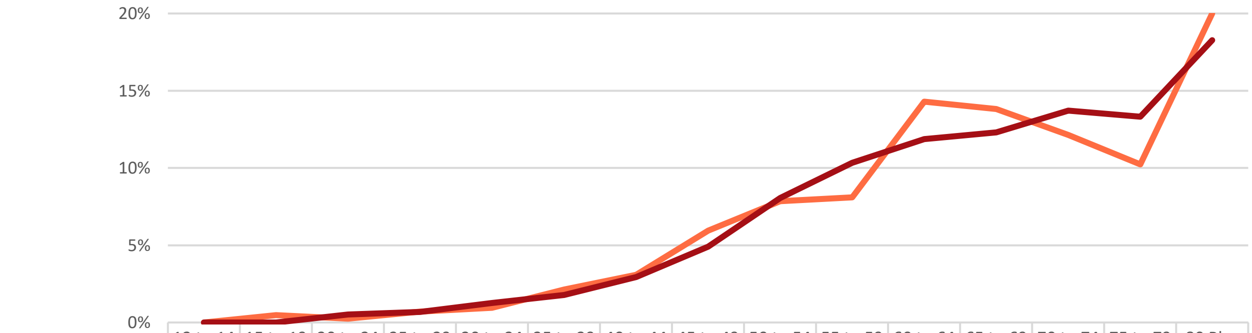

	12 to 14	15 to 19	20 to 24	25 to 29	30 to 34	35 to 39	40 to 44	45 to 49	50 to 54	55 to 59	60 to 64	65 to 69	70 to 74	75 to 79	80 Plus
VA Female	0%	0%	0%	1%	1%	2%	3%	6%	8%	8%	14%	14%	12%	10%	20%
GBD Female	0%	0%	1%	1%	1%	2%	3%	5%	8%	10%	12%	12%	14%	13%	18%

Both (Males and Females)

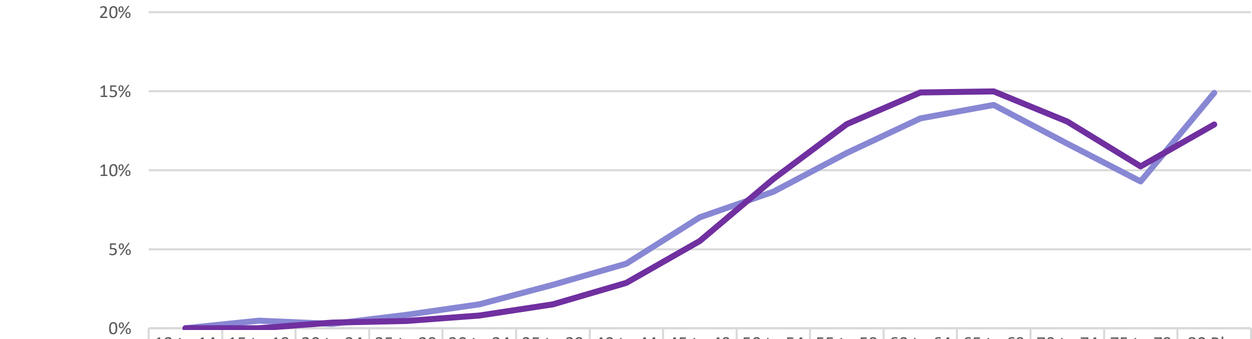

	12 to 14	15 to 19	20 to 24	25 to 29	30 to 34	35 to 39	40 to 44	45 to 49	50 to 54	55 to 59	60 to 64	65 to 69	70 to 74	75 to 79	80 Plus
VA Both	0%	0%	0%	1%	2%	3%	4%	7%	9%	11%	13%	14%	12%	9%	15%
GBD Both	0%	0%	0%	0%	1%	2%	3%	6%	9%	13%	15%	15%	13%	10%	13%

Falls

Falls	# VA Deaths
Male	459
Females	256
All (Male & Female)	715

Males

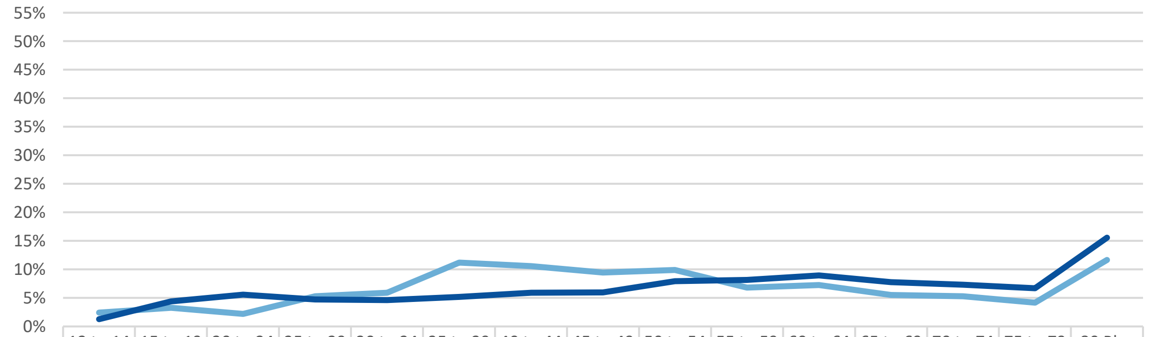

	12 to 14	15 to 19	20 to 24	25 to 29	30 to 34	35 to 39	40 to 44	45 to 49	50 to 54	55 to 59	60 to 64	65 to 69	70 to 74	75 to 79	80 Plus
VA Male	2%	3%	2%	5%	6%	11%	11%	9%	10%	7%	7%	5%	5%	4%	12%
GBD Male	1%	4%	6%	5%	5%	5%	6%	6%	8%	8%	9%	8%	7%	7%	16%

Females

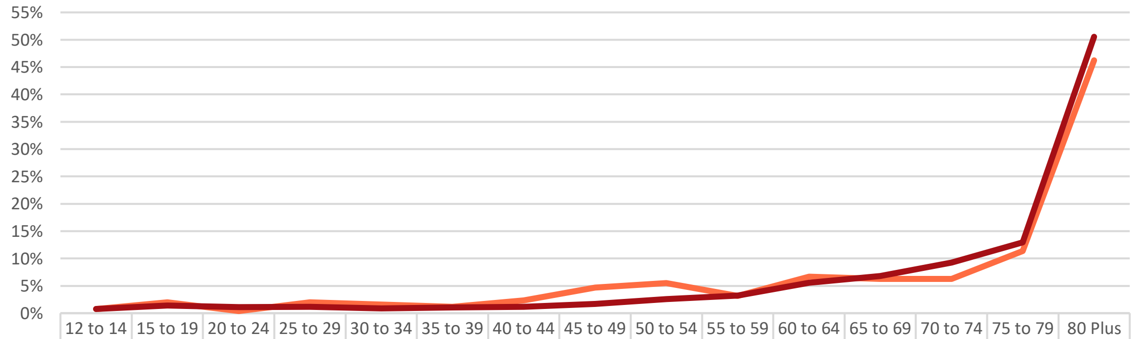

	12 to 14	15 to 19	20 to 24	25 to 29	30 to 34	35 to 39	40 to 44	45 to 49	50 to 54	55 to 59	60 to 64	65 to 69	70 to 74	75 to 79	80 Plus
VA Female	1%	2%	0%	2%	2%	1%	2%	5%	5%	3%	7%	6%	6%	11%	46%
GBD Female	1%	1%	1%	1%	1%	1%	1%	2%	3%	3%	6%	7%	9%	13%	51%

Both (Males and Females)

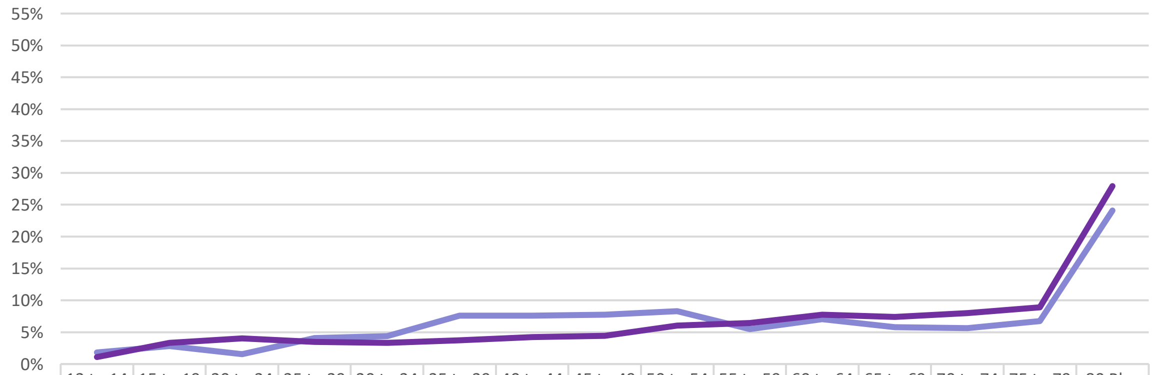

	12 to 14	15 to 19	20 to 24	25 to 29	30 to 34	35 to 39	40 to 44	45 to 49	50 to 54	55 to 59	60 to 64	65 to 69	70 to 74	75 to 79	80 Plus
VA Both	2%	3%	2%	4%	4%	8%	8%	8%	8%	5%	7%	6%	6%	7%	24%
GBD Both	1%	3%	4%	3%	3%	4%	4%	4%	6%	6%	8%	7%	8%	9%	28%
