# Supplementary figures and images for: Estimating causes of community death of adults in Myanmar from a nationwide population sample: Application of verbal autopsy
Source: PLOS Glob Public Health. 2023 Nov 1;3(11):e0002426. doi: 10.1371/journal.pgph.0002426 (PMC10619871; doi:10.1371/journal.pgph.0002426)

**S3 Fig: Age distribution of Undetermined cause of death (2018/2019 VA data combined)**

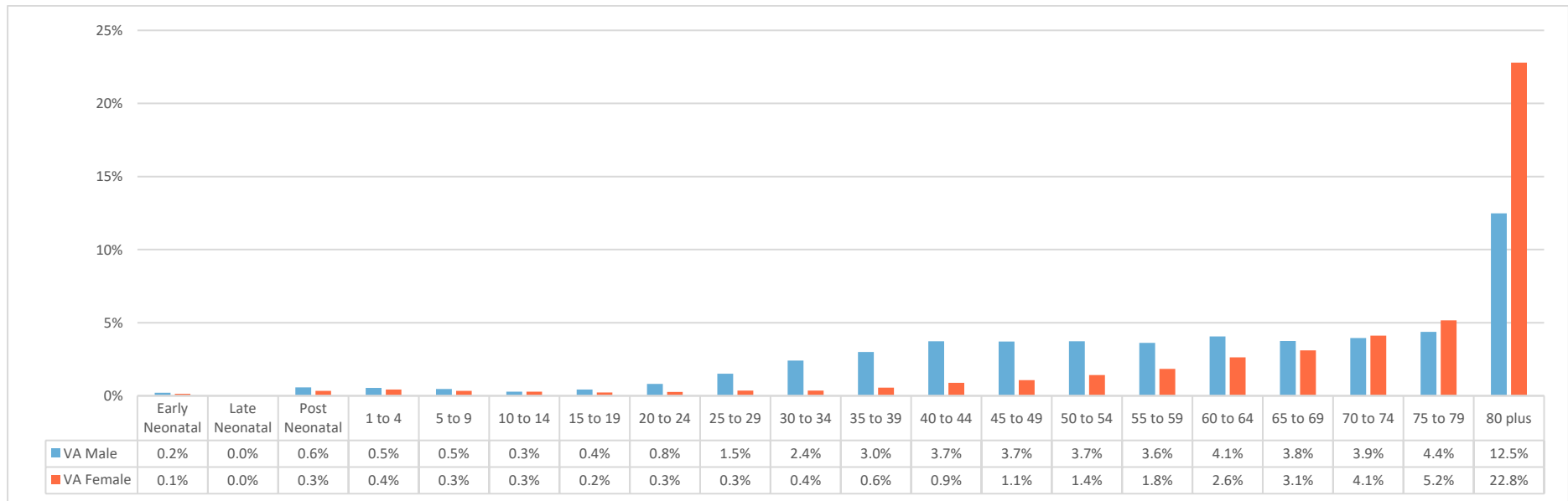

Supplement: S3 Fig — (PDF) [file pgph.0002426.s009.pdf]
