# Supplementary material for: Estimating causes of community death of adults in Myanmar from a nationwide population sample: Application of verbal autopsy
Source: PLOS Glob Public Health. 2023 Nov 1;3(11):e0002426. doi: 10.1371/journal.pgph.0002426 (PMC10619871; doi:10.1371/journal.pgph.0002426)
Supplement: Attachment — Submitted filename: Response to Reviewers.docx [file pgph.0002426.s010.docx]

Response to reviewers.

We thank the reviewers for taking the time to read the manuscript and provide important feedback. We provide a point-by-point response below and have made relevant adjustments to the manuscript as indicated. Line numbers below refer to the ‘Revised manuscript with track changes’.

Reviewer #1: • Data availability: The framing of the text that data cannot be fully made available means independent review of this data in the future is not possible. I would suggest that, it should be rephrased that the VA data is owned by Government and anyone wishing to access this data would need permission from the civil registration and vital statistics systems of the Government of Myanmar.

*Response: We thank the reviewer for their suggestion. We have rephrased the Data availability section to be: “The data used in this study are from the civil registration and vital statistics system of Myanmar. The data are owned by the Government of Myanmar, who apply restrictions to the availability and use of these data. The data in this study were used with the permission of the Government of Myanmar. Anyone wishing to access this data would need permission from the civil registration and vital statistics system of the Government of Myanmar. Hence, we are unable to make the data fully available for this manuscript.”*

• Conclusion: The focus of this study is on adults (12years+ by definition), why would VA data of neonates and children further improve policy utilization of VA data? What data from this research supports this position/recommendation?

*Response: We agree with the reviewer that this was mis-worded. We have rephrased the conclusion [lines 339-341] to note that the incompleteness VA for the child and neonatal age-group categories meant that we were not able to conduct a meaningful COD analysis. Improving VA completeness for child and neonates would allow this analysis of cause of death patterns in these age-groups and improve the policy utility of the VA data for these age-groups.*

• Line 46: Note that 7/17 is not half: either use a little over 2 in 5, or over 40% or just put 41% requiring cause-specific mortality data.

*Response: Thank you for this suggestion. We have amended accordingly [line 46].*

• Lines 37, 40, 41, 43, 46, 49 and 54, the comma should be placed after the reference and not before to make it reader friendly. Do same across the entire manuscript.

*Response: Thank you for this suggestion. We have made the necessary adjustments.*

• Line 62: include the total sample population of township of which 42 were selected. It is not very clear how this sample of 42 were selected (sample methodology).

*Response: The total sample population size and its proportion of the total population of Myanmar is noted [lines 70-72]. Please let us know if we have misunderstood the first line of your comment.*

*Regarding sampling, we have provided more detail in this section [line 62-65] which we hope clarifies. Whilst the sampling was not random, it was representative of the national population in terms of age structure (% aged 65+) and U5MR [lines 62-69].*

• Since the defined sample age is 12+, it would be good to know how this age group is distributed demographically as the VA is representing this exact population especially in the study population/sample population.

*Response: Thank you for this important comment. We have provided details of the distribution of the population aged 12+ in supplementary material (S3 Table: Age distribution of population (age 12+) in 42 townships by sex) and referred to in the text [line 154-155).*

• Line 137: The study title relates to ‘’adults’’ with a cut off of 12+ years and so why include under-five mortality VA data? Note in line 127, stillbirths data was collected but removed from the dataset. Hence if children below 12years VA data was collected, then it should be clearly stated that this dataset were cleaned out prior to analysis for consistency. Another issue is about what the legal definition of ‘’adult’’ is in Myanmar as 12+ years is being used as ‘’adults’’ for ease of standardization and country by country comparison? If this will pose a problem, then the title can include VA among people ‘’12 years and above’’ can be used in place of ‘’adult’’.

*Response: In line 137 [relevant entry now line 144], U5MR is described as being used as an input into the completeness method cited. It is used to represent the level of mortality in a population.*

*Regarding stillbirth, we agree with the reviewer that this is extraneous information since we do not analyse deaths under 12+. We have therefore deleted reference to stillbirths in the manuscript.*

*Regarding the definition of ‘adult’ in this manuscript - we thank the reviewer for this comment and the chance to clarify. For verbal autopsy the WHO standard is to use 12+ for adults – in large part to capture young maternal deaths. We have added a clarifying comment regarding the age-ranges for the categories [lines 91-93] and added a reference to the WHO Verbal Autopsy Standards webpage.*

• Please provide reference for the statement from lines 268 to 272.

*Response: We have provided references regarding the link between risk factors and the diseases mentioned [line 276]. We provide a link to the GBD2019 publication [line 281].*

• Lines 300-303: rephrase the limitation because the objective of this study was about utility and feasibility of VA amongst 12+ and so making refences to neonates confuses the issues being studied. This clearly impacts on aspects of the conclusions in this study (line 329).

*Response: We thank the reviewer for this comment. VA information was collected on all deaths and we are interested in the disease pattern for all age-groups. Unfortunately, the incompleteness of data in the child and neonatal categories meant we were not able to do a meaningful analysis on this data – only for adult deaths. We have added a line in the last para of data quality checks and data analysis [line 150] and also rephrased the first para of the limitations [lines 311-313]*

• Why didn’t AIDS show in Table 4 for females but in figure 4a it is reflected as 5% among females 12-49yrs?

*Response: Thank you for your query. In Figure 4a we demonstrate that AIDS is a leading cause of death for younger females (12-49 yrs). Table 4 illustrates top causes for all females. Younger females constitute only 13% of all female VA deaths and the disease distribution for all females therefore follows more closely that of older women (Figures 4b and 4c) for which AIDS is not a top cause of death. We hope that answers your question.*

Reviewer #2: This article is a meaningful thesis that systematically introduces methods that can be applied in developing countries that lack population registration systems and health personnel.
The only weakness is the lack of information about the IRB.
Nevertheless, by introducing a detailed data collection method, the verbal autopsy method suggests the possibility of supplementing autograph statistics in developing countries that lack statistical systems.

*Response: We thank Reviewer 2 for their positive comments. As noted, VA in Myanmar was collected as part of a routine data collection system by midwives who are responsible for registering deaths, not as a separate study. As such, the government has used the data to publish cause of death statistics for 2018 and 2019 in the Myanmar Statistical Yearbook 2020. Therefore, formal ethics was not appropriate or sought. However, midwives were trained in appropriate data collection methods, including informed consent and, as for any officially collected information, data were kept securely for use by the Myanmar Central Statistical Organisation in compiling their statistics.*
